# Bivalent Inhibitors of Mannose-Specific Bacterial Adhesion: A Xylose-Based Conformational Switch to Control Glycoligand Distance †

**DOI:** 10.3390/molecules30153074

**Published:** 2025-07-23

**Authors:** Sven Ole Jaeschke, Ingo vom Sondern, Thisbe K. Lindhorst

**Affiliations:** Otto Diels Institute of Organic Chemistry, Christiana Albertina University of Kiel, 24098 Kiel, Germany

**Keywords:** carbohydrate chemistry, carbohydrate recognition, conformational switch, bacterial adhesion, FimH, lectin inhibitors, inter-ligand distance, molecular modeling

## Abstract

Functional glycomimetics is suited to study the parameters of carbohydrate recognition that forms the basis of glycobiology. It is particularly attractive when a glycoligand allows for the investigation of two different states, such as varying distance between multiple glycoligands. Here, a xylopyranoside was employed as a scaffold for the presentation of two mannoside units which are ligands of the bacterial lectin FimH. The chair conformation of the central xyloside can be switched between a ^4^*C*_1_ and a ^1^*C*_4_ conformation whereby the two conjugated mannoside ligands are flipped from a di-equatorial into a di-axial position. Concomitantly, the distance between the two glycoligands changes and, as a consequence, so does the biological activity of the respective bivalent glycocluster, as shown in adhesion–inhibition assays with live bacteria. Molecular modeling was employed to correlate the inter-ligand distance with the structure of the formed glycocluster–FimH complex. Our study suggests that conformational switches can be employed and further advanced as smart molecular tools to study structural boundary conditions of carbohydrate recognition in a bottom-up approach.

## 1. Introduction

The carbohydrate-specific adhesion of *E. coli* bacteria to glycosylated surfaces is an important topic in glycoscience that arouses interest in two respects. First, bacterial adhesion is a prerequisite for infectious diseases and for biofilm formation. Knowing the involved mechanisms and being able to prevent the adhesion of bacteria, for example with carbohydrate-based inhibitors, can contribute to progress in diagnostics and therapy [1,2,3]. Second, bacterial adhesion can serve as a relevant biosystem with which to probe the parameters of carbohydrate recognition in a bottom-up approach using bespoke glycomimetics [4,5,6,7].

To accomplish adhesion to the glycocalyx of their host cells, bacteria use proteinaceous adhesive organelles, so-called fimbriae that project from the bacterial cell surface in multiple copies. Fimbriae have various carbohydrate specificities. Type 1 fimbriae are the best-known and most often studied fimbriae displaying a specificity for α-D-mannosides and are mediated by a lectin domain at the fimbrial tips called FimH [8]. Many different glycomimetics have been designed as FimH antagonists aiming at the inhibition of type 1 fimbriae-mediated bacterial adhesion [9,10,11,12]. It has become clear that various structural aspects govern the inhibitory power of carbohydrate-based inhibitors. These include the nature of the aglycone portion of a mannoside, which can exert favorable interactions at the entrance of the FimH carbohydrate recognition domain (CRD), specifically with the so-called tyrosine gate formed by amino acid residues Tyr48 and Tyr137 [13]. Hence, mannosides with aromatic aglycon moieties are especially powerful inhibitors of FimH as they can exert π-π interactions with the tyrosine gate [14]. Additionally, multivalency effects have often been observed with bivalent and multivalent glycoclusters [15,16]. Also, the distance between multiple mannoside ligands has been recognized as an important parameter in carbohydrate recognition, for example when glycoligands were studied, which are based on a rigid biphenyl scaffold [17,18,19].

**Figure 1:**
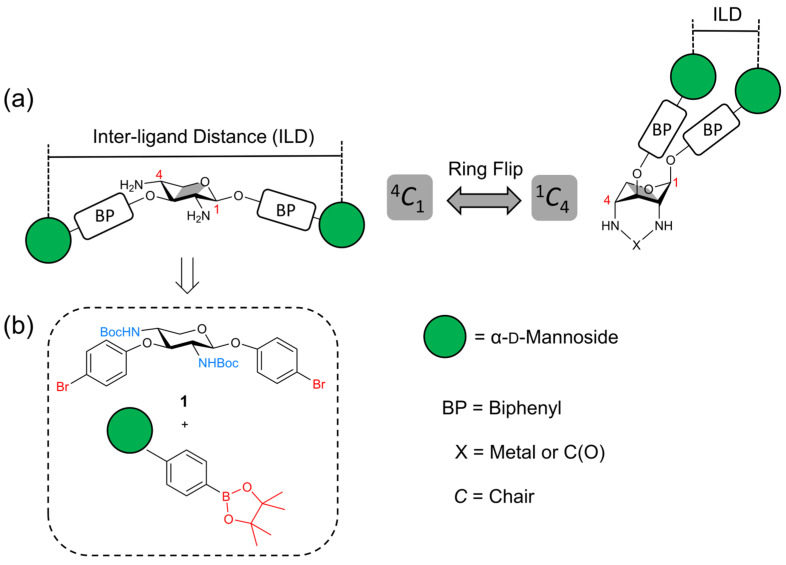
(**a**) The idea behind this account consists of switching the inter-ligand distance (ILD) within a bivalent glycocluster through the ring flip of a central xyloside scaffold. When the xyloside scaffold exists in the ^4^*C*_1_ conformation, the ILD is maximal whereas it is reduced when the xylopyranoside ring is forced into the ^1^*C*_4_ conformation. Additionally, in the ^1^*C*_4_ conformer the two biphenyl moieties can exert intramolecular π-π interactions which further contribute to the tight spatial organization of ligand presentation. (**b**) The retrosynthetic analysis of such a conformational switch leads back to the xyloside **1** which can be cross-coupled with a carbohydrate phenylboronic acid. Mannoside moieties are depicted as green circles according to the symbol nomenclature for glycans (SNFG) [20].

Here, we introduce a new type of bivalent inhibitor of type 1 fimbriae-mediated bacterial adhesion. The special feature of this type of glycocluster is that the distance between the two glycoligands can be varied through a conformational switch that is represented by a central xylopyranoside scaffold. Two glycoligand moieties which are attached to positions 1 and 3 of a xyloside scaffold differ greatly in their distance from each other depending on the conformation of the central sugar ring. This is so because the ring flip between a ^4^*C*_1_ and ^1^*C*_4_ xylose chair conformation forces a maximal orientational change of the ring substituents from all-equatorial to all-axial. Hence, while the two equatorially positioned glycoligand units at C-1 and C-2 point away from each other in the ^4^*C*_1_ conformation of the xyloside, they come much closer together in the ^1^*C*_4_ chair (Figure 1a).

The design of this “switchable” glycoligand is based on a 2,4-diamino xylopyranoside which has been employed previously to realize flipping between the two complementary chair conformations through metal complexation [21,22,23]. We have recently demonstrated that the iodosulfonamidation of a suitable glycal is the method of choice to achieve substituted 2,4-diamino xyloside stereo- and regioselectively [24]. Following this synthetic route, the substituted *para*-bromophenyl xyloside **1** was produced [24] (Figure 1b). It offers two features which are essential for the herein described approach of controlling glycoligand distance through conformational switching. First, the Boc-protected amino functions at positions 2 and 4 of the xyloside ring allow the switching of the pyranose ^4^*C*_1_ conformation into the flipped ^1^*C*_4_ conformation after removal of the Boc-protecting groups. This can be accomplished, e.g., through metal complexation of the liberated 2,4-diamine or through the covalent locking of the ^1^*C*_4_ conformation by formation of a cyclic urea derivative (vide infra, Scheme 2). Second, the *para*-bromophenyloxy residues at positions 1 and 3 of the sugar ring provide the option to ligate an arbitrary glycoligand that is functionalized with a phenylboronic acids moiety in a Pd-catalyzed cross-coupling reaction.

This approach enables the synthesis of the targeted bivalent glycoligands carrying two biphenyl mannoside moieties as ligands of the bacterial lectin FimH where the inter-ligand distance will vary depending on the chair conformation of the central xyloside scaffold, ^4^*C*_1_ or ^1^*C*_4_, respectively. The biphenyl linker units were chosen on purpose for several reasons. First, biphenyl α-D-mannopyranosides are known as high-affinity ligands of FimH [25,26]; second, their rigidity facilitates direct transfer of the spatial effect of the ring flip of the central xyloside to the relative orientation of the ligated mannoside ligands. Finally, in the glycocluster based on the xyloside in ^1^*C*_4_ conformation, the glycoligands can undergo intramolecular π-π interactions stabilizing a close inter-ligand distance [22].

Using a bivalent glycoligand based on such a conformational switch allows, firstly, the determination of how differential distances between two ligands affect the inhibitory potency of the corresponding compound. Second, the principle of a conformational switch can be explored which is attractive since such glycomimetics have not been used in carbohydrate recognition studies so far.

Hence, in this account we describe the synthesis of two bivalent glycoligands based on a switchable xylopyranoside scaffold in the ^4^*C*_1_ and in ^1^*C*_4_ conformation, respectively, and their investigation as inhibitors of type 1 fimbriae-mediated adhesion of *E. coli* on mannan-coated microtiter plates. Moreover, molecular dynamics and molecular modeling simulations were performed to provide a basis for the interpretation of the observed structure–function relationships.

## 2. Results

### 2.1. Synthesis

**Scheme 1:**
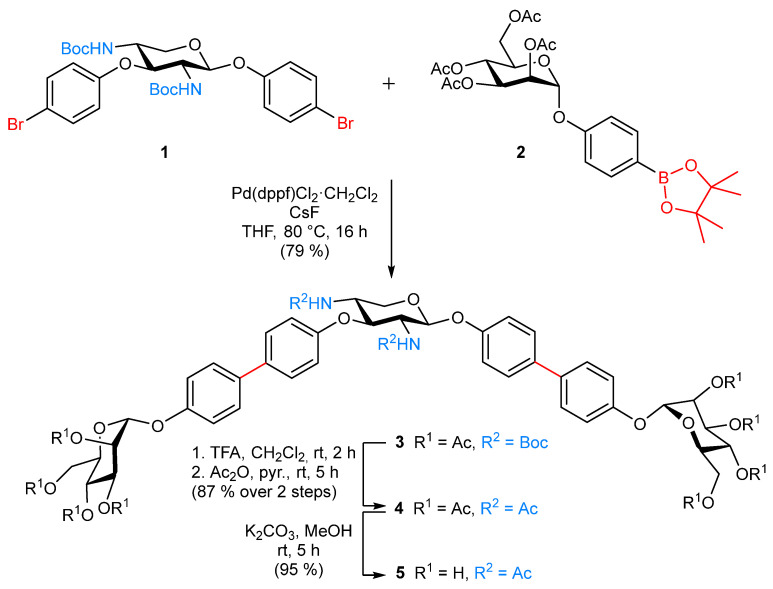
Synthesis of the divalent biphenylmannoside glycoclusters **3**, **4**, and **5**. The central xyloside scaffold unit in **4** and **5** adopts the ^4^*C*_1_ conformation (see text). The key step of this synthesis is a double Suzuki–Miyaura cross-coupling of the bis-arylhalide **1** and the phenylboronic acid **2**. TFA: trifluoroacetic acid.

Synthesis started with the *N*-Boc-protected 2,4-diamino xyloside **1** [24] in which the two *para*-bromophenyloxy residues at positions 1 and 3 of the sugar ring were intended for ligation with a suitable phenylboronic acids moiety in a Suzuki–Miyaura cross-coupling reaction [27]. Here, we employed the literature-known 4-mannopyranosyloxyphenyl boronate **2** [28,29], which can be obtained from the respective bromophenyl mannoside by standard procedures. In order to achieve the divalent biphenylmannoside glycocluster **3**, the Suzuki–Miyaura cross-coupling reaction was first attempted with Pd(PPh_3_)_4_ as the catalyst and Cs_2_CO_3_ or K_3_PO_4_ as the base (Scheme 1) [5]. However, no product was formed under these conditions. Also, variation of the temperature or the solvent did not lead to success. However, it was recognized that the xyloside **1** reacted with the Pd catalyst in an oxidative addition reaction, whereas the transmetallation and reductive elimination reaction steps did not occur. Because for these latter steps of the cross-coupling reaction the base is important, we next focused on the addition of fluorides. Fluorides are potent bases for Suzuki–Miyaura reactions due to the fluorophilicity of the organoboranes [30]. Indeed, when cesium fluoride (CsF) was employed as the base [31], cross-coupling of **1** and **2** in THF for 8 h at 80 °C using Pd(dppf)Cl_2_·CH_2_Cl_2_ as the catalyst led to the desired product **3**, however only in a moderate yield of 40%. By increasing the reaction time to 16 h, the yield was almost doubled to 79% (Scheme 1).

The investigation of the target glycocluster **3** by ^1^H NMR spectroscopy indicated a conformational equilibrium between various conformations of the xylopyranoside ring owing to line broadening of the signals. An analogous observation has been reported earlier for the xyloside **1** [24] and the occurring conformational dynamic has been further analyzed in a study combining spectroscopic and computational data [32]. Here however, two defined conformers, one in a ^4^*C*_1_ and the other in a ^1^*C*_4_ conformation, are needed in order to alter the distance between the scaffolded biphenyl mannoside ligands in a controlled way. Therefore, the *N*-protecting groups were exchanged in an attempt to omit the conformational dynamic of the xyloside scaffold. Removal of the Boc-protecting groups with TFA furnished the unprotected diamine, which was subsequently acetylated in the same pot to give the *N*-acetylated mannosyloxybiphenyl xyloside **4** in a yield of 87% over two steps. Note that free amino groups had to be avoided as they can lead to false results in biological testing. The ^1^H NMR-spectroscopic analysis of **4** then indicated a clear ^4^*C*_1_ conformation for the xyloside scaffold (as well as for the mannoside ligands) due to sharp and well-resolved peaks in the spectrum (see Supplementary Materials). The same was true for the OH-free xyloside-based glycocluster **5** which was obtained after removal of the *O*-acetyl groups under Zemplén conditions [33] in 95% yield (Scheme 1).

**Scheme 2:**
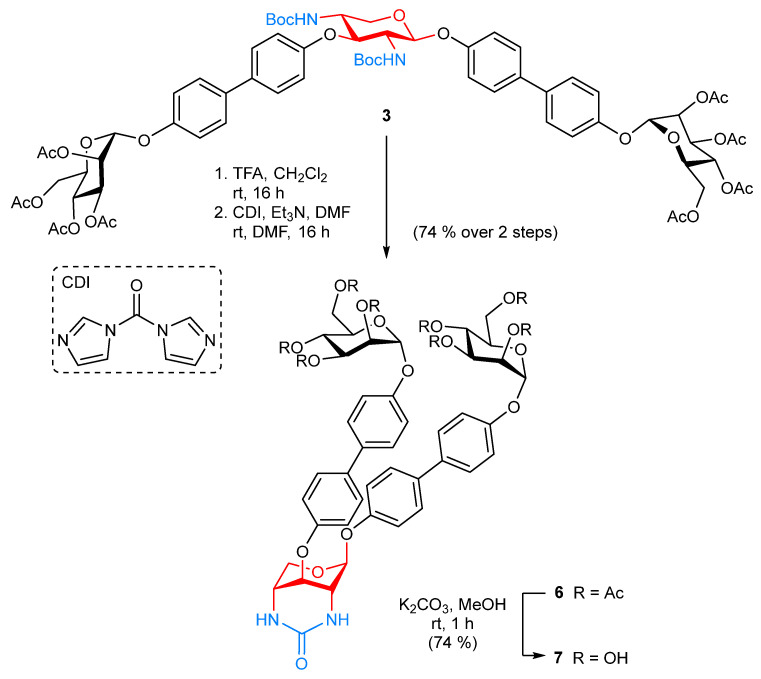
Synthesis of the divalent biphenylmannoside glycocluster **7** in which the central xyloside scaffold is locked in the ^1^*C*_4_ conformation. CDI: carbonyldiimidazole.

The biphenylmannoside glycocluster **5** is suitable as a divalent FimH antagonist in biological testing (vide infra). Here, the two biphenyl mannoside ligands are scaffolded on a xylopyranoside ring in ^4^*C*_1_ conformation. In order to achieve the complementary FimH antagonist in which the two biphenyl mannoside ligands are differently oriented, that is arranged on a xylopyranoside ring in ^1^*C*_4_ conformation, the ring flip of the central carbohydrate had to be achieved. To this end, the 2- and 4-amino groups were incorporated into a cyclic urea with an electrophilic C1 reagent. This reaction forces the xylopyranoside scaffold to adopt the ^1^*C*_4_ conformation. Starting from the bis-Boc-protected glycocluster **3**, first the Boc-protecting groups were cleaved with TFA and then the unprotected intermediate was subjected to an intramolecular cyclization reaction with carbonyldiimidazole (CDI) in the presence of Et_3_N in DMF [34]. Under these conditions, the bicyclic urea derivative **6** was obtained in 74% yield over two steps (Scheme 2). Its global deprotection under Zemplén conditions gave the target glycocluster **7** in which the two biphenyl mannoside ligands are differently oriented compared to **5** owing to the ring flip of the central xyloside hinge.

To obtain a feeling for the distance distribution of the two glycoligands conjugated to the central scaffold xyloside in **5** and **7**, molecular dynamics studies were carried out.

### 2.2. Molecular Dynamics

At first glance, the two biphenyl mannoside units in **5** and **7** seem to project from the xyloside scaffold very differently. However, there is quite some conformational freedom due to free rotation around the connecting single bonds which might lead to a different picture in reality. To assess the actual distance between the two mannose ligands in both glycoclusters, they were subjected to a molecular dynamics (MD) simulation. For this, the program Desmond [35,36] as implemented in the Schrödinger Maestro software package 2021-1 [37] was employed. The MD simulations were carried out in explicit water (SPC model [38] at 310 K for 200 ns; see Supplementary Materials). For both glycoclusters, the respective chair conformations (^4^*C*_1_ for **5** and ^1^*C*_4_ for **7**) of the central xyloside ring were assumed to remain stable over the simulation period. On the other hand, the spatial orientation of the biphenyl linker units is flexible during the simulation, influencing the distance between the glycosidically bound mannosyl residues.

**Figure 2:**
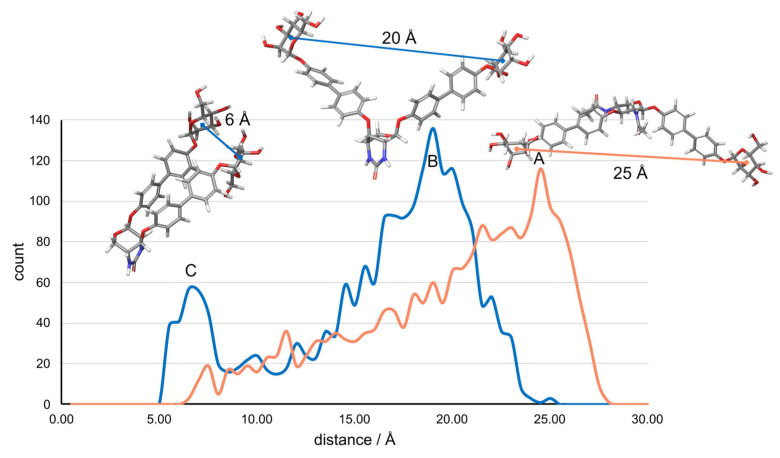
Probability distribution of inter-ring distances between the mannoside ligands scaffolded in **5** (bronze) and **7** (blue), respectively, and their occurrence as determined by molecular dynamics. Distances are measured between the centers of the mannopyranoside rings. Structures A, B, and C are representative snapshots of the MD simulation, illustrating the consequences of the conformational change of the central xylopyranoside chair from ^4^*C*_1_ (in **5**, bronze) to ^1^*C*_4_ (in **7**, blue). π-π interactions between the biphenyl moieties, which are axially aligned in **7**, result in structures like C. Structures rendered with Schrödinger. Color code for the structures: carbons (grey), nitrogens (blue), oxygen (red).

The obtained MD trajectories were analyzed to evaluate the distance between the centres of the two mannopyranoside rings projecting from the xyloside scaffold in **5** and **7** (Supplementary Material Figure S2). In compound **5**, the inter-ring distance fluctuated between 7 and 27 Å, while in compound **7**, it ranged from 5 to 25 Å. To characterize the most frequently occurring distances, the probability distribution of the distances between the two mannoside ligands were plotted for **5** and **7** (Figure 2). It can be seen that the change of the conformation of the central xyloside ring from ^4^*C*_1_ to ^1^*C*_4_ clearly impacts the amplitude of the inter-ligand distances as well as their distribution. However, the differences are less pronounced than intuitively expected. In glycocluster **5**, the distance between the two mannoside rings reaches a maximum in comparison to **7** owing to the ^4^*C*_1_ conformation of the xyloside scaffold and the conformational flexibility of the biphenyl units (Figure 2, structure A). Here, the most frequently occurring distance is ~25 Å. On the contrary, the inter-ligand distances in **7** are smaller due to the ^1^*C*_4_ conformation of the xyloside scaffold. Note that two main populations are seen in **7**. One population shows an inter-ligand distance amplitude at ~20 Å (Figure 2, structure B), whereas in the other population (Figure 2, structure C), the two mannoside ligands are very close to each other (~6 Å) due to π-π interactions between the biphenyl units. This is a phenomenon which has been seen before in arylether-substituted 2,4-diamino xyloside switches where π-π stacking can lead to stabilization of the ^1^*C*_4_ conformation of the xyloside [22].

### 2.3. Biological Testing

Although the inter-ligand distances in glycoclusters **5** and **7** cover a relatively wide range (cf. Section 2.2), in the most frequently occurring conformers the distance between the biphenyl mannoside antennas is different (~25 Å for **5** and ~20 and ~6 Å for **7**). In order to test if this difference, which is a result of the inverse ring conformation of the central xyloside scaffold, effects the properties of **5** and **7** as bivalent FimH ligands, an adhesion–inhibition assay with type 1 fimbriated *E. coli* bacteria (PKL1162) [39] was performed. In the literature-known assay [40], adhesion of GFP (green fluorescent protein)-transfected *E. coli* to a mannan-coated surface is determined (for details see Supplementary Materials). Remaining adhered bacteria after inhibition and washing can be determined by fluorescence read-out. The glycoclusters **5** and **7** were employed in serial dilutions on microtiter plates where they compete with the mannan coating for FimH binding. As a result, dose-response inhibition curves are obtained (Supplementary Material Figure S1) of which IC_50_ values for each inhibitor can be deduced (see Supplementary Material Table S1). Since biological experiments and IC_50_ values can vary significantly between independent experiments with live bacteria, methyl α-D-mannopyranoside (MeMan) was tested on the same plate serving as internal reference inhibitor. The inhibitory potencies of the tested glycoclusters were then referenced to MeMan leading to so-called relative inhibitory potencies (RIP values). Since the solubility of the glycoclusters **5** and **7** in aqueous buffer is limited due to the included hydrophobic biphenyl moieties, 5% DMSO were added to the glycocluster solutions (0.4 mm). This does, however, not compromise the assay.

The results of the adhesion–inhibition assay are summarized in Table 1. As the biphenyl moieties glycosidically linked to the mannoside ligands can exert favorable interactions with the tyrosine gate at the entrance of the FimH CRD, their FimH affinity is considerably higher than that of MeMan [41]. The resulting RIP values of **5** and **7** were in the range of literature-known aryl mannoside ligands (cf. Supplementary Materials) [42]. However strikingly, the inhibitory potencies of the glycoclusters **5** and **7** differ from each other by a factor of close to 3 in spite of the fact that both inhibitors display two copies of exactly the same biphenyl α-D-mannopyranoside ligand.

As the differences seen in the inhibitory potencies between **5** and **7** cannot be attributed to the nature of the ligand or the aglycon moiety of the mannoside, nor to the valency of the tested glycocluster (both are bivalent), it seems likely that the conformational dynamics of the glycoligand antennas and, more specifically, the various inter-ligand distances displayed in **5** and **7** are decisive for the different ligand properties. In **5**, the inter-ligand distance is maximal and this seems to be favorable for the inhibitory potency of the respective glycocluster. Even binding of two FimH proteins by a bivalent glycoligand has been described in the literature [43], however, it is not sure, that the same binding mode is happening in an assay with live type 1 fimbriated bacteria. Nevertheless, a greater conformational availability of glycoligands such as in **5** apparently facilitates lectin binding leading to higher FimH affinity (lower IC_50_ values). In glycocluster **7**, the mannoside ligands span shorter inter-ligand distances resulting in lower lectin affinity. Especially those conformers occurring in **7** where the two glycoligands stack to each other through π-π interactions (Figure 2, structure C) can be assumed to show a relatively weak FimH affinity as any of the two the mannoside ligands is poorly available for complexation within the CRD. This interpretation is supported by an analogous observation described in the literature [44].

The biological assays show that the inter-ligand distance in a bivalent glycocluster together with the conformational availability of the glycoligands displayed in the respective inhibitor impact the affinity for a specific lectin. Note, that in the herein described approach, this difference is caused by the flip between the ^4^*C*_1_ and ^1^*C*_4_ chair conformation of a central carbohydrate scaffold. The idea behind this approach bears the potential to modulate and control, respectively, ligand affinity by switching carbohydrate conformation.

In order to rationalize the measured effects, the interactions of the glycoclusters with the bacterial lectin FimH were further studied by molecular modeling.

### 2.4. Molecular Modeling

To investigate the structure of the glycocluster–FimH complexes, docking studies were performed with the tested glycoclusters **5** and **7** and the lectin FimH using the software Glide 2024-2 [45] as implemented in the Schrödinger software package 2024-2 [46] (see Supplementary Materials for details). The tyrosine residue Tyr48 as part of the FimH tyrosine gate shows a pronounced flexibility [25,47,48,49]. Consequently, depending on the complexed ligand, varying conformations of the tyrosine gate were observed. As the open gate conformation can favorably stabilize complexes with Man ligands carrying an aromatic aglycon moiety, the respective FimH structure (PDB code 6G2S) [50] was employed in the modeling. The top scoring results obtained in the docking experiments for both glycoclusters were subjected to a MM-GBSA calculation [51] (molecular mechanics energies combined with generalized born and surface area continuum solvation) to yield binding energies for both FimH ligands, **5** and **7**. The docking scores and corresponding binding energies are collected in Table 2. While the docking scores are similar for both glycoclusters, the binding energies differ which is in consistency with the different RIP values measured in the bioassays.

**Figure 3:**
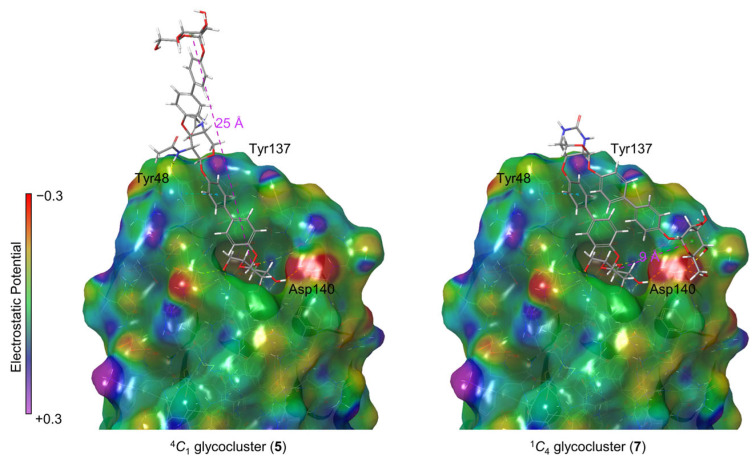
Connolly surface representations of FimH–glycocluster complexes as derived from molecular docking using the open gate conformation of FimH (pdb: 6G2S) [50]. The xylose-scaffolded glycoclusters **5** and **7** are depicted as stick models. Protein coloring represents the electrostatic potential of the surface (positive charges in purple, neutral in green, negative charges in red, cf. depicted color bar). Hydrogen bonds, π-π interactions, and inter-ring distance are indicated as yellow, blue, and purple dashed lines, respectively.

Docking showed that in both inspected cases, an α-D-mannoside moiety is buried within the FimH CRD interacting through the typical hydrogen bond network [1]. Both glycoclusters show explicit π-π interactions between the biphenyl aglycone of the mannoside ligand and Tyr48 (light blue dotted lines in Figure 3), which are responsible for a high inhibitory potency in comparison to MeMan (cf. Table 1). The docking studies suggest, that glycocluster **5** with the central xyloside in ^4^*C*_1_ conformation binds to FimH such that the anomeric mannoside unit occupies the FimH CRD, while the second mannoside ligand conjugated to the 3-position of the xyloside scaffold protrudes outwards, being poised for potential multivalent interactions with another FimH domain in close proximity (Figure 3) [43]. In addition, the FimH-**5** complex is further stabilized by a hydrogen bond between the amido group at position 2 of the xyloside scaffold and the Tyr48 residue of FimH. The inter-ligand distance between the two mannoside glycoligands in the complex is 25 Å, paralleling with the results from the MD simulation (Section 2.2).

In contrast, in the bivalent glycocluster **7**, where the xyloside scaffold is locked in a ^1^*C*_4_ conformation, the mannoside ligand that is conjugated with the 3-position of the scaffold is complexed within the FimH CRD. Here, the glycocluster adopts an angled conformation owing to the ^1^*C*_4_ conformation of the xyloside ring which is flipped in comparison to **5**. The ^1^*C*_4_ conformation of the central sugar also results in a shorter inter-ligand distance of about 9 Å in the ligand–FimH complex (cf. Section 2.2). Additional secondary interactions are observed for the anomeric mannoside unit forming hydrogen bonds with the polar amino acid residue Asp140. Here, the more restricted structure of the molecule most likely precludes the option for an effective multivalent interaction of the glycocluster. Hence, the simulated FimH–glycocluster complexes show differences between glycoclusters **5** and **7** which can be related to the reduced inhibitory potency (lower RIP value) of glycocluster **7** in comparison to glycocluster **5**.

## 3. Materials and Methods

### 3.1. General Information Regarding Synthesis and Spectroscopy

Moisture-sensitive reactions were carried out in flame-dried glassware and under a positive pressure of nitrogen. Analytical thin layer chromatography (TLC) was performed on silica gel plates (GF 254, Merck, Darmstadt, Germany). Visualization was achieved by UV light and/or with 10 % sulfuric acid in ethanol, vanillin (3.0 g vanillin and 0.5 mL H_2_SO_4_ in 100 mL EtOH) or ninhydrin, followed by heat treatment at approx. 200 °C. The products were purified by flash chromatography on silica gel columns (Merck, 230–400 mesh, particle size 0.040–0.063 mm, Darmstadt, Germany) or by automated flash chromatography using a puriFlash 450 device from the Interchim^®^ company (Montluçon, France). MeOH was dried over magnesium under a nitrogen atmosphere. Optical rotations were measured with a PerkinElmer 241 polarimeter (PerkinElmer, Waltham, MA, USA) with a sodium D-line (589 nm) and a cuvette of 10 cm path length, in the solvents indicated. Proton (^1^H) nuclear magnetic resonance spectra and carbon (^13^C) nuclear magnetic resonance spectra were recorded on a Bruker DRX-500 and AV-600 spectrometer (Bruker, Billerica, MA, USA) at 300 K. Chemical shifts are referenced to the internal standard tetramethylsilane (TMS) or to the residual proton of the NMR solvent. Multiplets (multiplicity s = singlet, d = doublet, t = triplet, m = multiplet) are listed according to chemical shift, coupling constants are given in Hertz (Hz). Full assignment of the signals was achieved by using 2D NMR techniques (^1^H-^1^H COSY, ^1^H-^13^C HMBC and ^1^H-^13^C HSQC). Infrared (IR) spectra were measured with a PerkinElmer FT-IR Paragon 1000 (ATR) spectrometer (PerkinElmer, Waltham, MA, USA) and are reported in cm^−1^. ESI mass spectra were recorded on a LCQ Classic from Thermo Finnigan (Somerset, NJ, USA).

### 3.2. Synthesis

*4′-[(1,1′-Biphenyl)-2,3,4,6-tetra-O-acetyl-α-d-mannopyranoside]-4-yl 2,4-dideoxy-2,4-N-Boc-3-O-{4′-[(1,1′-biphenyl)-2,3,4,6-tetra-O-acetyl-α-d-mannopyranoside]-4-yl}-β-d-xylopyranoside* (**3**). The xyloside **1** [26] (60.0 mg, 87.2 µmol), the pinacoyl ester **2** [28] (144 mg, 261 µmol), CsF (79.4 mg, 523 µmol) and Pd(dppf)Cl_2_·CH_2_Cl_2_ (14.2 mg, 17.4 µmol) were dissolved in dry THF (6.00 mL) and the reaction mixture was heated to 80 °C and stirred for 16 h. After cooling to room temperature, it was diluted with ethyl acetate and the organic layer was washed with H_2_O. The combined organic layers were dried over MgSO_4_, it was filtered and concentrated. Purification by column chromatography on silica gel (cyclohexane/ethyl acetate, 1:1) yielded **3** (93.0 mg, 79%) as a colorless solid; R_f_ = 0.22 (cyclohexane/ethyl acetate, 1:1); [α${]}_{D}^{23}$ = +31.2 (c 0.02, acetone). ^1^H NMR (600 MHz, acetone-d_6_, 298 K, TMS): δ = 7.63–7.57 (m, 6H, 6 H_arom_), 7.52 (d, ^3^J = 8.7 Hz, 2H, H_arom_), 7.27–7.21 (m, 4H, 4 H_arom_), 7.20–7.11 (m, 4H, 4 H_arom_), 6.50 (d, ^3^J_NH,2_ = 8.6 Hz, 1H, NH), 6.33 (d, ^3^J_NH,4_ = 7.9 Hz, 1H, NH), 5.71 (d, ^3^J_1,2_ = 1.1 Hz, 1H, H-1_Man_), 5.70 (d, ^3^J_1,2_ = 1.1 Hz, 1H, H-1_Man_), 5.51–5.46 (m, 4H, 2 H-2_Man_, 2 H-3_Man_), 5.43 (d, ^3^J_1,2_ = 7.2 Hz, 1H, H-1_Xyl_), 5.34 (dd~t, ^3^J_3,4_ = 9.9 Hz, ^3^J_4,5_ = 9.9 Hz, 2H, 2 H-4_Man_), 4.93 (t, ^3^J_3,4_ = 9.2 Hz, ^3^J_2,3_ = 9.2 Hz, 1H, H-3_Xyl_), 4.25 (dd, ^2^J_6a,6b_ = 12.0 Hz, ^3^J_5,6a_ = 5.9 Hz, 1H, H-6a_Man_), 4.24 (dd, ^2^J_6a,6b_ = 12.0 Hz, ^3^J_5,6a_ = 5.9 Hz, 1H, H-6a_Man_), 4.20–4.15 (m, 2H, 2 H-5_Man_), 4.10–4.07 (m, 2H, 2 H-6b_Man_), 4.03 (dd, ^2^J_5a,5b_ = 11.1 Hz, ^3^J_5a,4_ = 4.7 Hz, 1H, H-5a_Xyl_), 3.96–3.90 (m, 1H, H 4_Xyl_), 3.84–3.78 (m, 1H, H-2_Xyl_), 3.73 (t, ^2^J_5a,5b_ = 10.9 Hz, ^3^J_5a,4_ = 10.9 Hz, 1H, H-5b_Xyl_), 2.16, 2.06, 1.99, 1.95, 1.94 (each s, 24H, 8 C(O)CH_3_), 1.33, 1.31 (each s, 18H, 2 C(CH_3_)_3_) ppm. ^13^C NMR (125 MHz, acetone-d_6_, 298 K, TMS): δ = 170.62, 170.40, 170.37, 170.28 (8C, C(O)CH_3_), 160.33 (NH-C(O)), 158.03 (NH-C(O)), 155.91 (C_quart_), 155.75 (C_quart_), 138.50 (2 C_quart_), 136.50 (C_quart_), 136.54 (C_quart_), 136.27 (C_quart_), 135.53 (C_quart_), 128.61 (2 CH_arom_), 128.57 (2 CH_arom_), 128.47 (2 CH_arom_), 128.24 (2 CH_arom_), 118.24 (2 CH_arom_), 118.23 (2 CH_arom_), 118.15 (2 CH_arom_), 118.12 (2 CH_arom_), 100.54 (C-1_Xyl_), 96.97 (2 C-1_Man_), 79.06 (C-3_Xyl_), 70.28 (2 C-5_Man_), 69.88 (2 C-2_Man_), 69.80 (2 C-3_Man_), 66.61 (2 C-4_Man_), 64.64 (C-5_Xyl_), 62.94 (2 C-6_Man_), 57.89 (C-2_Xyl_), 53.26 (C-4_Xyl_), 28.55 (3C, C(CH_3_)_3_), 28.53 (3C, C(CH_3_)_3_), 21.71, 20.65, 20.61, 20.59 (8C, C(O)CH_3_) ppm. IR (ATR) ν_max_/cm^−1^ = 3309, 1750, 1683, 1497, 1367, 1220, 1039, 824, 601; ESI-HRMS *m*/*z* = 1362.5278 [M + NH_4_]^+^, calcd for [M + NH_4_]^+^ 1362.5292.
*4′-[(1,1′-Biphenyl)-2,3,4,6-tetra-O-acetyl-α-d-mannopyranoside]-4-yl 2,4-dideoxy-2,4-N-acetyl-3-O-{4′-[(1,1′-biphenyl)-2,3,4,6-tetra-O-acetyl-α-d-mannopyranoside]-4-yl}-β-d-xylopyranoside* (**4**). The N-Boc-protected glycocluster **3** (20.0 mg, 14.9 µmol) was dissolved in CH_2_Cl_2_ (1.00 mL), TFA (200 µL) was added and the reaction mixture stirred for 2 h at room temperature. Then all volatiles were removed in vacuo and the residue co-evaporated with toluene (3×). The crude product was dissolved in dry pyridine (2.00 mL), Ac_2_O (500 µL) was added and the reaction mixture stirred at room temperature for 5 h. Then it was diluted with ethyl acetate and the organic layer was subsequently washed with 1N HCl, sodium bicarbonate and brine. The organic layer was dried over MgSO_4_, its was filtered and concentrated. Purification by column chromatography on silica (toluene/acetone, 1:1) gave the title compound **4** (16.0 mg, 87%) as a colorless solid; R_f_ = 0.32 (toluene/acetone, 1:1); [α${]}_{D}^{23}$ = +16.6 (c 0.02, acetone). ^1^H NMR (500 MHz, acetone-d_6_, 298 K, TMS): δ = 7.63–7.59 (m, 4H, 4 H_arom_), 7.58–7.54 (m, 4H, 4 H_arom_), 7.51 (d, ^3^J_NH,2_ = 8.6 Hz, 1H, NH), 7.36 (d, ^3^J_NH,4_ = 8.4 Hz, 1H, NH), 7.27–7.22 (m, 4H, 4 H_arom_), 7.19 (d, ^3^J = 8.9 Hz, 2H, H_arom_), 7.11 (d, ^3^J = 8.8 Hz, 2H, H_arom_), 5.71–5.69 (m, 2H, 2 H-1_Man_), 5.57 (d, ^3^J_1,2_ = 6.5 Hz, 1H, H-1_Xyl_), 5.50-5.46 (m, 4H, 2 H-2_Man_, 2 H-3_Man_), 5.33 (dd~t, ^3^J_3,4_ = 9.9 Hz, ^3^J_4,5_ = 9.9 Hz, 2H, 2 H-4_Man_), 4.99 (t, ^3^J_3,4_ = 7.9 Hz, ^3^J_2,3_ = 7.9 Hz, 1H, H-3_Xyl_), 4.23 (dd, ^2^J_6a,6b_ = 12.0 Hz, ^3^J_5,6a_ = 5.9 Hz, 2H, 2 H-6a_Man_), 4.21–4.10 (m, 4H, 2 H-5_Man_, H-5a_Xyl_, H-4_Xyl_), 4.08 (dd, ^2^J_6a,6b_ = 12.0 Hz, ^3^J_5,6b_ = 2.3 Hz, 2H, 2 H-6b_Man_), 4.05–3.99 (m, 1H, H-2_Xyl_), 3.73 (dd, ^2^J_5a,5b_ = 11.6 Hz, ^3^J_5a,4_ = 8.4 Hz, 1H, H-5b_Xyl_), 2.16, 2.05, 1.99, 1.94 (each s, 24H, 8 C(O)CH_3_), 1.80, 1.75 (each s, 6H, NHC(O)CH_3_) ppm. ^13^C NMR (125 MHz, acetone-d_6_, 298 K, TMS): δ = 170.65, 170.43, 170.40, 170.31 (8C, C(O)CH_3_), 170.54, 170.34 (2C, NHC(O)CH_3_), 159.70 (C_quart_), 159.62 (C_quart_), 155.94 (C_quart_), 155.80 (C_quart_), 136.45 (C_quart_), 136.31 (C_quart_), 134.33 (C_quart_), 128.63 (2 CH_arom_), 128.59 (2 CH_arom_), 128.49 (2 CH_arom_), 128.23 (2 CH_arom_), 118.28 (4C, CH_arom_), 118.10 (2 CH_arom_), 117.97 (2 CH_arom_), 99.43 (C-1_Xyl_), 97.01 (2 C-1_Man_), 77.09 (C-3_Xyl_), 70.31 (2 C-5_Man_), 69.9 2 (2 C-2_Man_), 69.83 (2 C-3_Man_), 66.64 (2 C-4_Man_), 63.38 (C-5_Xyl_), 62.97 (2 C-6_Man_), 55.57 (C-2_Xyl_), 50.93 (C-4_Xyl_), 23.09, 23.00 (2C, NHC(O)CH_3_), 21.74, 20.68, 20.64, 20.61 (8C, C(O)CH_3_) ppm. IR (ATR) ν_max_/cm^−1^ = 2298, 1750, 1662, 1496, 1370, 1221, 1039, 825, 600. ESI-HRMS *m*/*z* = 1246.4428 [M + NH_4_]^+^, calcd for [M + NH_4_]^+^ = 1246.4449.
*4′-[(1,1′-Biphenyl)-α-d-mannopyranosyloxy]-4-yl 2,4-dideoxy-2,4-N-acetyl-3-O-{4′-[(1,1′-biphenyl)-α-d-mannopyranosyloxy]-4-yl}-β-d-xylopyranoside* (**5**). The protected glycocluster **4** (13.0 mg, 10.6 µmol) was dissolved in MeOH (1.00 mL), K_2_CO_3_ (414 µg, 3.00 µmol) was added and it was stirred for 5 h at room temperature. It was neutralized with Amberlite^®^ IR-120, filtered and concentrated to dryness. Co-evaporation with toluene and CH_2_Cl_2_ yielded **5** (9.00 mg, 95%) as a colorless solid; [α${]}_{D}^{23}$ = +26.3 (c 0.02, MeOH). ^1^H NMR (600 MHz, MeOD-d_3_, 298 K, TMS): δ = 7.53–7.47 (m, 8H, 8 H_arom_), 7.24–7.05 (m, 8H, 8 H_arom_), 5.51 (s, 2H, 2 H-1_Man_), 5.35 (d, ^3^J_1,2_ = 7.7 Hz, 1H, H-1_Xyl_), 4.85–4.82 (m, 1H, H-3_Xyl_), 4.24–4.18 (m, 1H, H-4_Xyl_), 4.06–4.00 (m, 4H, H-2_Xyl_, H-5a_Xyl_, 2 H-2_Man_), 3.92 (dd, ^3^J_3,4_ = 8.3 Hz, ^3^J_2,3_ = 4.0 Hz, 2H, 2 H-3_Man_), 3.80–3.70 (m, 6H, 2 H-4_Man_, 2 H-6a_Man_, 2 H-6b_Man_), 3.66–3.59 (m, 3H, 2 H-5_Man_, H-5b_Xyl_), 1.81, 1.76 (each s, 6H, NHC(O)CH_3_) ppm. ^13^C NMR (125 MHz, MeOD-d_3_, 298 K, TMS): δ = 173.68, 173.53 (2C, NHC(O)CH_3_), 159.96 (C_quart_), 157.96 (C_quart_), 157.23 (C_quart_), 157.14 (C_quart_), 136.70 (C_quart_), 136.11 (C_quart_), 136.06 (C_quart_), 135.69 (C_quart_), 129.22 (2 CH_arom_), 128.77 (2 CH_arom_), 128.64 (2 CH_arom_), 128.61 (2 CH_arom_), 118.21 (4C, CH_arom_), 118.11 (4C, CH_arom_), 100.47 (C-1_Xyl_), 100.23 (2 C-1_Man_), 78.89 (C-3_Xyl_), 75.40 (2 C-5_Man_), 72.43 (2 C-2_Man_), 72.03 (2 C-3_Man_), 68.36 (2 C-4_Man_), 64.40 (C-5_Xyl_), 62.70 (2 C-6_Man_), 57.20 (C-2_Xyl_), 52.18 (C-4_Xyl_), 22.74, 22.66 (2C, NHC(O)CH_3_) ppm. IR (ATR) ν_max_/cm^−1^ = 3364, 2467, 1494, 1220, 999, 824, 677, 575. ESI-HRMS *m*/*z* = 910.3593 [M + NH_4_]^+^, calcd for [M + NH_4_]^+^ 910.3604.
*4′-[(1,1′-Biphenyl)-2,3,4,6-tetra-O-acetyl-α-d-mannopyranoside]-4-yl 2,4-N-carbonyl-2,4-dideoxy-3-O-{4′-[(1,1′-biphenyl)-2,3,4,6-tetra-O-acetyl-α-d-mannopyranoside]-4-yl}-β-d-xylopyranoside* (**6**). The glycocluster **3** (20.0 mg, 14.9 µmol) was dissolved in CH_2_Cl_2_ (1.00 mL), TFA (200 µL) was added and it was stirred for 2 h at room temperature. Then all volatiles were removed under reduced pressure and the residue was co-evaporated with toluene (3×). The crude product was dissolved in DMF (150 µL) and N,N-carbonyldiimidazole (2.90 mg, 17.9 µmol) was added, followed by dropwise addition of triethylamine (10.4 µL, 74.5 µmol). The reaction mixture was stirred for 16 h at room temperature, then it was diluted with ethyl acetate and washed with 1N HCl, sodium bicarbonate and brine. The combined organic layer were dried over MgSO_4_, it was filtered and concentrated. Purification by column chromatography on silica gel (toluene/acetone, 4:6) gave the title compound **6** (13.0 mg, 74%) as a colorless solid; R_f_ 0.15 (toluene/acetone, 4:6); [α${]}_{D}^{23}$ = −27.3 (c 0.02, acetone). ^1^H NMR (600 MHz, acetone-d_6_, 298 K, TMS): δ = 7.68 (d, ^3^J = 8.7 Hz, 2H, 2 CH_arom_), 7.65 (d, ^3^J = 8.7 Hz, 2H, 2 CH_arom_), 7.59 (d, ^3^J = 8.7 Hz, 2H, 2 CH_arom_), 7.55 (d, ^3^J = 8.7 Hz, 2H, 2 CH_arom_), 7.29 (d, ^3^J = 8.7 Hz, 2H, 2 CH_arom_), 7.26 (d, ^3^J = 8.7 Hz, 2H, 2 CH_arom_), 7.23 (d, ^3^J = 8.7 Hz, 2H, 2 CH_arom_), 7.09 (d, ^3^J = 8.7 Hz, 2H, 2 CH_arom_), 6.17 (d, ^3^J_NH,2_ = 4.2 Hz, 1H, NH), 6.07 (d, ^3^J_NH,4_ = 4.6 Hz, 1H, NH), 5.72–5.68 (m, 2H, 2 H-1_Man_), 5.52 (s, 1H, H-1_Xyl_), 5.51–5.45 (m, 4H, 2 H-2_Man_, 2 H-3_Man_), 5.33 (dd~t, ^3^J_3,4_ = 10.0 Hz, ^3^J_4,5_ = 10.0 Hz, 2H, 2 H-4_Man_), 5.06 (t, ^3^J_3,4_ = 3.1 Hz, ^3^J_2,3_ = 3.1 Hz, 1H, H-3_Xyl_), 4.45 (d, ^2^J_5a,5b_ = 11.4 Hz, 1H, H-5a_Xyl_), 4.23 (dd, ^2^J_6a,6b_ = 11.9 Hz, ^3^J_5,6a_ = 5.9 Hz, 2H, 2 H-6a_Man_), 4.20–4.14 (m, 2H, 2 H-5_Man_), 4.08 (dd, ^2^J_6a,6b_ = 12.0 Hz, ^3^J_5,6b_ = 2.2 Hz, 2H, 2 H-6b_Man_), 3.95 (s, 1H, H-4_Xyl_), 3.69 (s, 1H, H-2_Xyl_), 3.60 (d, ^2^J_5a,5b_ = 12.0 Hz, 1H, H-5b_Xyl_), 2.16, 2.16, 2.06, 2.04, 1.99, 1.99, 1.95, 1.93 (each s, 24H, 8 C(O)CH_3_) ppm. ^13^C NMR (125 MHz, acetone-d_6_, 298 K, TMS): δ = 170.66, 170.43, 170.40, 170.31 (8 C(O)CH_3_), 157.37, 157.22, 155.86, 155.82, 136.35, 136.31, 134.81, 134.59 (8 C_quart_), 128.71, 128.53, 128.52, 128.49, 118.27, 118.23, 117.57, 117.32 (16 CH_arom_), 99.31 (C-1_Xyl_), 96.98, 96.96 (2 C-1_Man_), 70.26 (2 C-5_Man_), 69.88 (2 C-2_Man_), 69.80 (2 C-3_Man_), 68.88 (C-3_Xyl_), 66.60 (2 C-4_Man_), 62.94 (2 C-6_Man_), 61.98 (C-5_Xyl_), 48.57 (C-2_Xyl_), 47.85 (C-4_Xyl_), 21.71, 20.65, 20.61, 20.59 (8 C(O)CH_3_) ppm. IR (ATR) ν_max_/cm^−1^ = 2925, 1749, 1607, 1496, 1369, 1217, 1039, 825, 598. ESI-HRMS *m*/*z* = 1188.4013 [M + NH_4_]^+^, calcd for [M + NH_4_]^+^ 1188.4031.
*4′-[(1,1′-Biphenyl)-α-d-mannopyranoside]-4-yl 2,4-N-carbonyl-2,4-dideoxy-3-O-{4′-[(1,1′-biphenyl)-α-d-mannopyranoside]-4-yl}-β-d-xylopyranoside* (**7**). The protected glycocluster **6** (10.0 mg, 8.54 µmol) was dissolved in MeOH (1.00 mL), K_2_CO_3_ (354 µg, 2.56 µmol) was added and the reaction mixture was stirred for 4 h at room temperature. It was neutralized with Amberlite^®^ IR-120 (Sigma-Aldrich, St. Louis, MO, USA), filtered and concentrated to dryness. Co-evaporation with toluene and CH_2_Cl_2_ gave **7** (5.30 mg, 74%) as a colorless solid; [α${]}_{D}^{23}$ = +36.2 (c 0.02, MeOH). ^1^H NMR (600 MHz, MeOD-d_3_, 298 K, TMS): δ = 7.63–7.46 (m, 8H, 8 H_arom_), 7.25–7.01 (m, 8H, 8 H_arom_), 5.52 (s, 1H, H-1_Man_), 5.50 (s, 1H, H-1_Man_), 5.43 (s, 1H, H-1_Xyl_), 5.01 (t, ^3^J_3,4_ = 3.7 Hz, ^3^J_2,3_ = 3.7 Hz, 1H, H-3_Xyl_), 4.50 (dd, ^2^J_5a,5b_ = 11.5 Hz, ^3^J_4,5a_ = 11.5 Hz, H, H-5a_Xyl_), 4.04-4.01 (m, 2H, 2 H-2_Man_), 3.94-3.90 (m, 2H, 2 H-3_Man_), 3.89 (s, 1H, H-2_Xyl_), 3.80-3.57 (m, 10H, 2 H-4_Man_, 2 H-5_Man_, 2 H-6a_Man_, 2 H-6b_Man_, H-4_Xyl_, H-5b_Xyl_), ppm. ^13^C NMR (125 MHz, MeOD-d_3_, 298 K, TMS): δ = 160.15 (NHC(O)NH), 157.41 (C_quart_), 157.36 (C_quart_), 157.19 (C_quart_), 157.14 (C_quart_), 136.18 (C_quart_), 136.15 (C_quart_), 136.00 (C_quart_), 135.85 (C_quart_), 128.96 (2 CH_arom_), 128.75 (2 CH_arom_), 128.72 (2 CH_arom_), 128.68 (2 CH_arom_), 118.14 (2C, CH_arom_), 118.08 (2C, CH_arom_), 117.79 (2C, CH_arom_), 117.46 (2C, CH_arom_), 99.64 (C-1_Xyl_), 100.24 (2 C-1_Man_), 75.39(2 C-5_Man_), 72.43 (2 C-3_Man_), 72.03 (2 C-2_Man_), 68.68 (C-3_Xyl_), 68.36 (2 C-4_Man_), 62.69 (2 C-6_Man_), 61.94 (C-5_Xyl_), 52.79 (C-4_Xyl_), 47.85 (C-2_Xyl_) ppm. IR (ATR) ν_max_/cm^−1^ = 3251, 2013, 1495, 1220, 999, 825, 674, 584. ESI-HRMS *m*/*z* = 852.3178 [M + NH_4_]^+^, calcd for [M + NH_4_]^+^ 852.3185.

### 3.3. Cultivation of Bacteria

GFP-expressing *E. coli* bacteria (strain PKL1162, see Supplementary Materials) were cultured in 5.00 mL LB medium and incubated overnight at 37 °C and 100 rpm. Afterwards the mixture was centrifuged at 4 °C and 5000 rpm for 15 min. The bacteria pellet was washed twice with PBS buffer (2.00 mL) and then resuspended in PBS buffer. Finally, the suspension was adjusted to OD600 = 0.4.

### 3.4. Adhesion–Inhibition Assay with GFP-PKL1162 E. coli Bacteria

Inhibitor solutions of the respective glycosides **5** and **7** (0.4 mM in PBS buffer) as well as methyl α-D-mannopyranoside (MeMan, 200 mM in PBS buffer) were prepared and serial dilutions (1:2, 10 steps) of each solution added to the mannan-coated microtiter plates (50 µL/well, see Supplementary Materials). Next, the prepared bacterial suspension (OD_600_ = 0.4, 50 µL/well) was added and the microtiter plates were incubated at 37 °C and 100 rpm for 45 min. The plates were washed with PBS buffer (3 × 150 µL/well) and then the wells were filled with PBS (100 µL/well) and the fluorescence intensity (485 nm/535 nm) was determined. On each individual plate the standard inhibitor MeMan was tested in parallel. Each compound was tested in duplicates or triplicates, respectively.

### 3.5. Molecular Dynamics

The structures of **5** and **7** were built by using Maestro and minimized by use of MacroModel with the OPLS3 force field in implicit water (GB/SA continuum solvation model); for details, see the Supplementary Materials.

### 3.6. Molecular Modeling

For molecular modeling, the Schrödinger software package 2024-2 implementing the Maestro interface was used [46]; for details see the Supplementary Materials.

## 4. Conclusions

Carbohydrate recognition is governed by a multitude of parameters, among which the inter-ligand distance in a bivalent glycocluster is an important aspect. The idea behind this account has been to adjust the distance between two glycoligands by flipping the chair conformation of a central carbohydrate scaffold. This approach supplements a related concept that uses photoswitchable glycomimetics to alter the spatial orientation of glycoligands [7,52,53,54]. The herein introduced conformational switch is based on a known 2,4-diamino-xyloside, which can be flipped from a ^4^*C*_1_ into a ^1^*C*_4_ conformation through the chemical fixation of the two amino functions. Concomitantly, the distance between two biphenyl mannoside units conjugated to positions 1 and 3 of the central xyloside ring changes. In the ^4^*C*_1_ glycocluster (**5**) the most populated conformers show inter-ligand distances of ~25 Å, whereas the ^1^*C*_4_ glycocluster (**7**) the inter-ligand distances are shorter. Two populations can be seen in the case of **7** with most common inter-ligand distances of ~20 Å and ~6 Å, respectively.

As biphenyl mannosides are excellent ligands of the bacterial lectin FimH that mediates bacterial adhesion, both bivalent glycoclusters were tested as inhibitors of FimH-mediated adhesion of live *E. coli*. It turned out that the inhibitory potency of **5** is almost 3 times higher than that of **7**. This finding can be attributed to a difference in the distance between the glycoligands, since the glycoclusters **5** and **7** do not differ in any other structural aspect. Nevertheless, it is difficult to conclusively interpret the results of the bioassays, why molecular modeling studies were performed. Modeling revealed binding energies for the two carbohydrate-FimH complexes which parallel with the results from the adhesion–inhibition tests. In other words, the comparably higher FimH affinity of **5** can be correlated with its higher potency as inhibitor of mannose-specific bacterial adhesion. Furthermore, molecular modeling suggests that **5** is complexed within the FimH CRD such, that multivalent interactions with an adjacent lectin domain are possible, whereas this is excluded in the more densely packed structure of **7**.

In conclusion, modeling results rationalize the different RIP values measured for the bivalent glycoclusters **5** and **7** by highlighting how inter-ligand distance and the spatial orientation of ligands modulate both binding affinity and the potential for multivalent interactions. Furthermore, the idea of exploring differential carbohydrate binding by utilizing a conformational glycoswitch is a valuable new approach in glycoscience. We will further advance this concept also in order to shed more light on the so far underexplored dynamic processes in carbohydrate recognition.

## Figures and Tables

**Table 1:** Inhibitory potencies of the xylose-scaffolded glycoclusters **5** and **7** as determined in adhesion–inhibition assays with type 1 fimbriated *E. coli* bacteria (PKL1162).

Glycocluster	IC_50_ ^a^ [µmol]	IC_50_ MeMan ^b^ [mmol]	RIP ^c^	Average RIP ^d^
**5** ^4^*C*_1_ conformation of the xyloside scaffold	15.2 (±1.5)	7.08 (±0.44)	466 (±76)	410 (±68)
		
	15.7 (±1.3)	5.34 (±0.48)	339 (±60)	
**7** ^1^*C*_4_ conformation of the xyloside scaffold	63.9 (±14.1)	7.08 (±0.44)	111 (±31)	157 (±45)
		
	36.0 (±11.1)	5.34 (±0.48)	148 (±59)	

^a^ IC_50_ values are average values of duplicate or triplicate results on one plate. The fitting error from non-linear regression is given in brackets. ^b^ IC_50_ (MeMan) values are average values of triplicate results on the same plate. The fitting error from non-linear regression is given in brackets. ^c^ RIP values are based on the inhibitory potency of MeMan tested on the same microplate IP(MeMan) ≡ 1); RIP(glycocluster) = IC_50_(MeMan)/IC_50_(glycocluster). The fitting error from error propagation is given in brackets (see Supplementary Materials for details). ^d^ Average RIPs are mean values of two independent experiments (I and II) with error propagation in brackets (see Supplementary Materials for details).

**Table 2:** Computed results from molecular modeling studies with the synthetic ligands **5** and **7** and the open gate conformer of FimH (pdb: 6G2S) [50]. The best docking scores and corresponding binding energies (MM-GBSA) using Glide and Prime are listed. Lower values suggest higher FimH affinity. RIP values (higher values relating to stronger FimH binding) are shown for comparison.

Glycocluster (RIP)	Glide Score	Binding Energy [kcal mol^−1^]
^4^*C*_1_ glycocluster **5** (410 ± 68)	−10.468	−83.02
^1^*C*_4_ glycocluster **7** (157 ± 45)	−10.581	−67.85

## Data Availability

The data from this study are included in the article and Supplementary Materials.

## Supplementary Materials

The following supporting information can be downloaded at: https://www.mdpi.com/article/10.3390/molecules30153074/s1, Figure S1: Inhibition curves obtained with glycoclusters **5** and **7** as inhibitors of type 1 fimbriae-mediated bacterial adhesion to mannan; Table S1: IC_50_ values as deduced from the inhibition curves obtained with MeMan, ^4^C_1_ cluster **5** and ^1^C_4_ cluster **7** and corresponding RIP values; Figure S2: Distances between the Man-ligand residues as a function of the simulation time; Table S2: Scoring values for docking of glycoclusters **5** and **7** into the open gate (pdb: 6G2S) conformation of FimH using Glide; Table S3: Values of computed binding energies ∆G_Bind_ (in kcal mol^−1^) obtained from MM-GBSA calculations for **5** and **7** into the open gate (pdb: 6G2S) conformation of FimH; Figures S3–S12: ^1^H NMR and ^13^C NMR spectra of synthesized compounds. Reference [55] is cited in the supplementary materials.
