# Supplementary material for: Bivalent Inhibitors of Mannose-Specific Bacterial Adhesion: A Xylose-Based Conformational Switch to Control Glycoligand Distance
Source: Molecules. 2025 Jul 23;30(15):3074. doi: 10.3390/molecules30153074 (PMC12348410; doi:10.3390/molecules30153074)
Supplement: Supplementary file 1 [file molecules-30-03074-s001.zip › molecules-3752739-supplementary.pdf]

Supplementary Material

Bivalent Inhibitors of Mannose-Specific Bacterial Adhesion: A Xylose-Based Conformational Switch to Control Glycoligand Distance

Sven Ole Jaeschke, Ingo vom Sondern and Thisbe K. Lindhorst *

Otto Diels Institute of Organic Chemistry, Christiana Albertina University of Kiel, 24098 Kiel, Germany

* Correspondence: tklind@oc.uni-kiel.de

Contents

1. **Biological Testing**
2. **Molecular Modeling**
3. **NMR spectra of the synthesized compounds**

1. Biological testing

Equipment

The equipment was sterilized in an autoclave before usage. To determine the optical density of the bacterial suspension, a Jenway Spectrophotometer Model 7305 was used.

Bacteria

For the binding assays the GFP-expressing *E. coli* bacteria strain PKL1162, produced in the laboratory of Per Klemm, was used [1]. This *E. coli* strain PKL1162 was constructed by insertion of the plasmid pPKL1174 into the strain SAR18. The pPKL1174 plasmid contains the *fim* gene cluster, which is responsible for the expression of type 1 fimbriae. SAR18 includes the *gfp* gene in its genome, controlled by a constitutive promoter. The final bacterial strain PKL1162 expresses type 1 fimbriae as the only fimbriae type in addition to green fluorescence protein (GFP) allowing fluorescence read-out.

Buffers

For the biological testing the following buffers and media were used. All solutions were prepared with bidestilled water and were autoclaved before usage. The solutions were prepared by following procedures:

LB medium: Trypton (10.0 g), sodium chloride (10.0 g) and yeast extract (5.00 g) were dissolved in 1 L bidest. water and then adjusted to pH = 7.0. The solution was autoclaved and afterwards antibiotic (ampicillin (100 mg), chloramphenicol (50.0 mg)) were added.

PBS buffer: Sodium chloride (8.00 g), potassium chloride (200 mg), sodium biphosphate (1.44 g) and potassium biphosphate (200 mg) were dissolved in 1 L bidest. water and the pH value was adjusted to 7.2.

PBST buffer: Tween®20 (0.05% v/v) was added to PBS buffer.

Carbonate buffer (pH 9.6): sodium carbonate (1.59 g) and sodium hydrogen carbonate (2.52 g) were dissolved in distilled deionized water (1.00 L).

Mannan coating of microtiter plates

The published assay [2] was adapted and modified as follows: Black 96-well microtiter plates (NuncTM, Maxisorp®) were incubated with a solution of mannan from *Saccharomyces cerevisiae* (1.2 mg/mL in carbonate buffer, 120 µL/well) and desiccated overnight at 37 °C and 100 rpm. Then the plates were washed with PBST (3 x 150 µL/well) and blocked with polyvinyl alcohol (PVA) (1% in PBS, 120 µL/well) at 37 °C and 120 rpm for 2 h. Afterwards the microtiter plates were washed with PBST (3 x 150 µL/well).

Inhibition curves of the adhesion-inhibition assay with GFP expressing *E. coli* bacteria.

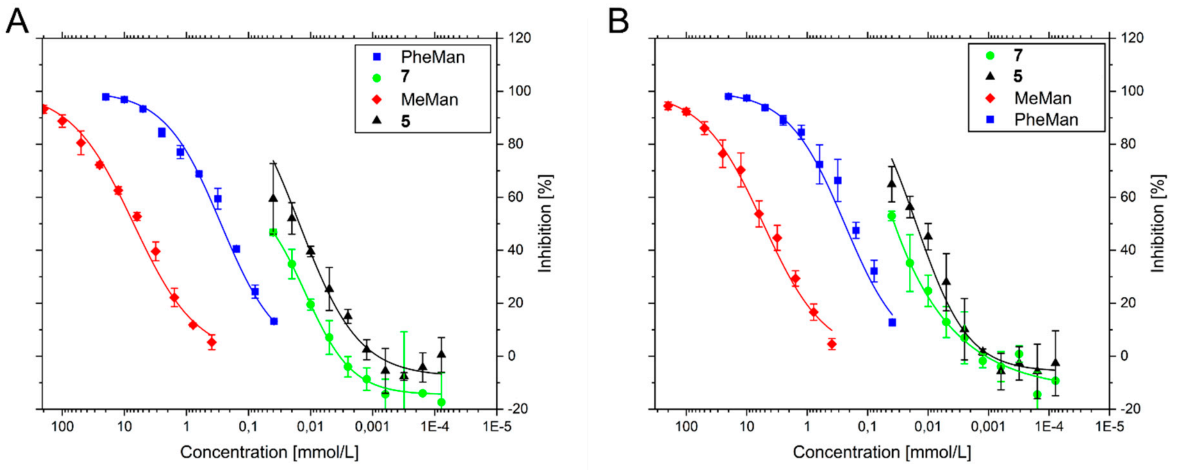

Figure S1. Inhibition curves obtained from two separate experiments A and B with glycoclusters **5** and **7** as inhibitors of type 1 fimbriae-mediated bacterial adhesion to mannan. MeMan was tested simultaneously on each plate. Sigmoidal concentration-response curves were fitted by non-linear regression. Error bars are standard deviations from triplicate values on one plate. MeMan: methyl α-D-mannopyranoside; PheMan: phenyl α-D-mannopyranoside.

Table S1. IC_{50} values as deduced from the inhibition curves obtained with MeMan, $^4C_1$ cluster **5** and $^1C_4$ cluster **7** and corresponding RIP values.

Plate	Results	MeMan	$^4C_1$ cluster (5)	$^1C_4$ cluster (7)
A	IC_{50} [a] [mmol]	7.08 (±0.44)	0.0152 (±0.0015)	0.0639 (±0.0141)
	RIP [b]	1.00	466 (±76)	111 (±31)
B	IC_{50} [a] [mmol]	5.34 (±0.48)	0.0157 (±0.0013)	0.0360 (±0.0111)
	RIP [b]	1.00	339 (±60)	148 (±59)
	average RIP [c]	1.00	410 (±68)	157 (±45)

[a] IC_{50} values are average values of at least duplicate results. Note that IC_{50} values can vary significantly in independent experiments with live bacteria.

[b] RIP values are based on the inhibitory potency of methyl α-D-mannopyranoside (MeMan), which was tested on the same microtiter plate (MeMan, IP = 1); RIP = IC_{50}(MeMan)/IC_{50}(tested compound). Errors are determined by error propagation with the following formula:

$$\mathbf{\Delta RIP} = \left|\frac{\mathbf{1}}{\mathbf{IC_{50}(glycan)}}\cdot\mathbf{\Delta IC_{50}(MeMan)}\right| + \left|-\frac{\mathbf{IC_{50}(MeMan)}}{\mathbf{IC_{50}(glycan)^2}}\cdot\mathbf{\Delta IC_{50}(glycan)}\right|$$

[c] Average RIP values of two independent experiments are given with error propagation using the following formula:

$$\mathbf{\Delta Mean\ RIP} = \frac{\mathbf{1}}{\mathbf{2}}\sqrt{(\mathbf{\Delta RIP_A})^2 + (\mathbf{\Delta RIP_D})^2}$$

2. Molecular modeling

For molecular modeling the Schrödinger software package implementing the Maestro interface was used [3,4].

Molecular dynamics

The structures of **5** and **7** were built by using Maestro and minimized by use of MacroModel, with the OPLS3 force field in implicit water (GB/SA continuum solvation model). Molecular dynamics in explicit water were carried out with the Desmond software, implemented in the Schrödinger package [5]. First, an orthorhombic solvent box (SPC H_2O molecules [6]), including **5** or **7**, with a buffering distance of 15 Å between the edges and the solute was generated. Each solvated system was then

subjected to a molecular dynamics simulation at 310 K for 200 ns (time step=100 fs, energy=1.2, NPT conditions), with monitoring of the geometry every ps. The trajectories were analyzed by using the “Simulation Event Analysis” tool, implemented in the Desmond program, in order to calculate the distances between the Man glycoligand portions of the inspected glycoclusters. Results for the ring distances are shown in Figure S2.

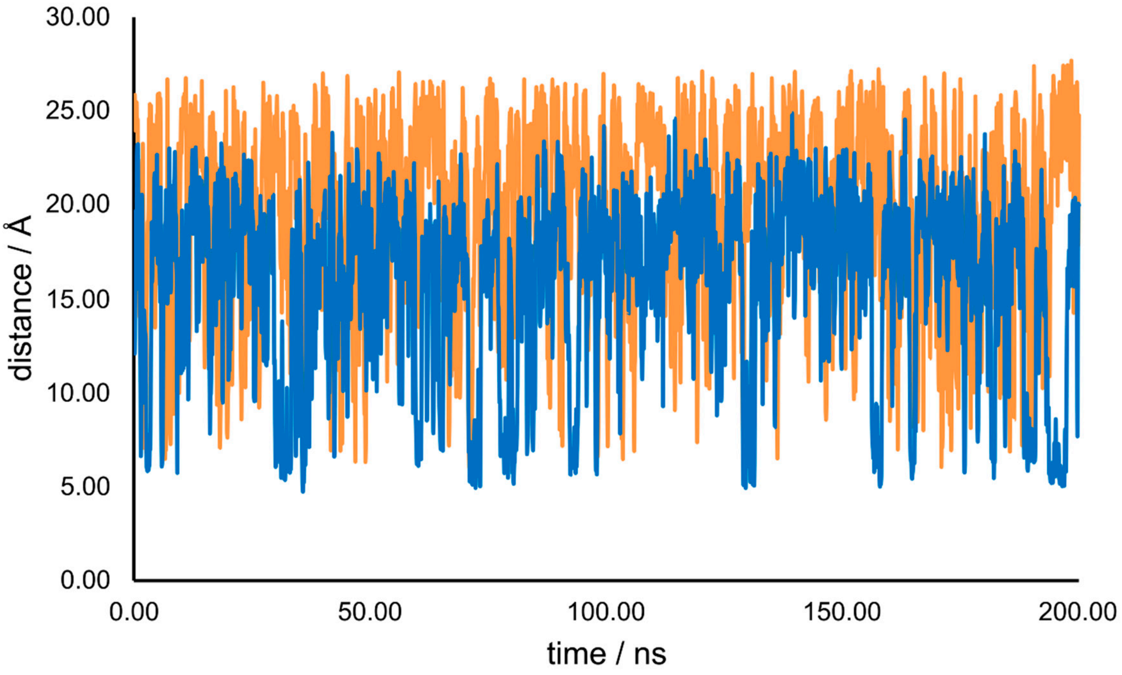

Figure S2: Distances between the glycoligand residues as a function of the simulation time. Distances of the $^4C_1$ cluster **5** are shown in bronze, distances of the $^1C_4$ cluster **7** in blue. The distances were measured between the centers of the α-D-mannopyranoside rings.

Glide docking

All ligands were built using Maestro and prepared for docking using LigPrep and the OPLS4 force field [7]. The crystal structure of FimH in its open gate form (pdb: 6G2S) [8] conformation was constructed using the protein preparation wizard implemented in Maestro.

Receptor grid for docking was built using Glide [9], by defining a box of 35x35x35 Å around the centroid of the ligand complexed within the binding site of the FimH crystal structure. Inside this box the ligand diameter midpoint of the docked ligand is required

to be. The receptor grid was further extended by 30 Å in all directions. Hydroxy groups of Tyr48 and Tyr137 were set rotatable. The receptor grid was built using the OPLS4 force field. Extra precision (XP) docking was carried out with Glide, setting the ligand sampling to flexible. Ring conformations were not sampled during docking. Nitrogen inversions were sampled during docking. Epic state penalties were added to docking scores. A constraint was put on the core structure to match the ligand structure in the known crystal structure (pdb 6G2S). At most, 20 poses were reported per ligand and a post-docking minimization was performed with a threshold for rejecting minimized poses of 0.50 kcal mol^{-1}. Additionally, poses with a RMSD less than 0.5 Å and a maximum atomic displacement less than 1.3 Å were treated as duplicates and discarded. Results are collected in Table S2.

Calculation of binding energies was performed by subjecting the top scoring binding poses from Glide docking to a MM-GBSA [10] (molecular mechanics generalized born surface area) calculation, giving the free binding energy ΔG_{Bind} in kcal mol-1. The MM-GBSA calculations were performed using the VGSB solvation model and the OPLS4 force field. Results are collected in Table S3.

Table S2. Scoring values for docking of glycoclusters **5** and **7** into the open gate (pdb: 6G2S) conformation of FimH using Glide.

Glycocluster	Docking Score	XP HBond	Glide evdw	Glide ecoul	Glide energy	Glide emodel
**5** ($^4C_1$)	-10.468	-6.336	-30.655	-22.793	-53.448	-88.994
**5** ($^4C_1$)	-10.256	-6.336	-30.569	-22.867	-53.437	-89.137
**5** ($^4C_1$)	-10.193	-6.336	-27.925	-26.418	-54.343	-86.93
**5** ($^4C_1$)	-9.932	-6.336	-30.561	-21.049	-51.61	-86.374
**5** ($^4C_1$)	-9.666	-6.336	-30.644	-20.977	-51.621	-86.292
**7** ($^1C_4$)	-10.581	-6.533	-36.745	-26.175	-62.92	-102.58
**7** ($^1C_4$)	-10.578	-6.533	-33.631	-29.59	-63.22	-103.825
**7** ($^1C_4$)	-10.259	-6.533	-32.563	-31.268	-63.83	-102.755
**7** ($^1C_4$)	-7.902	-6.533	-35.079	-24.695	-59.774	-100.849
**7** ($^1C_4$)	-7.741	-6.533	-35.105	-24.516	-59.622	-99.135

Table S3. Values of computed binding energies ΔG_{Bind} (in kcal mol^{-1}) obtained from MM-GBSA calculations for **5** and **7** into the open gate (pdb: 6G2S) conformation of FimH. Top scoring binding poses were selected.

Glyco-cluster	Docking Score	ΔG_{Bind}	$\Delta G_{Bind\_Coulomb}$	$\Delta G_{Bind\_Covalent}$	ΔG_{Bind_Hbond}	ΔG_{Bind_Lipo}	$\Delta G_{Bind\_Solv\_GB}$	$\Delta G_{Bind\_packing}$	ΔG_{Bind_vdW}	Ligand Strain Energy
**5** ($^4C_1$)	-10.468	-83.02	-49.81	12.71	-6.33	-25.18	25.92	-2.66	-37.66	13.025
**7** ($^1C_4$)	-10.581	-67.85	-34.28	7.48	-7.24	-20.23	28.21	-3.4	-38.39	27.125

3. NMR spectra of the synthesized compounds

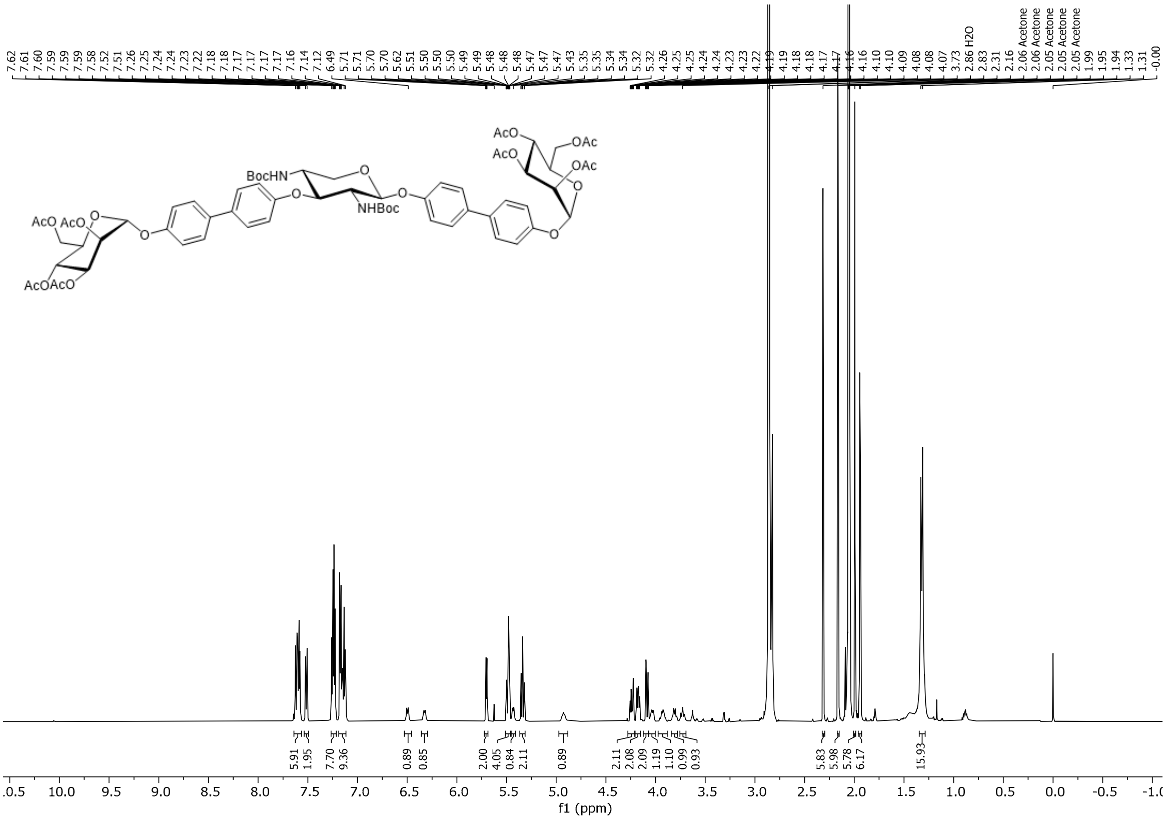

Figure S3. ^{1}H NMR spectrum of **3** (600 MHz, acetone-d$_6$, 298 K).

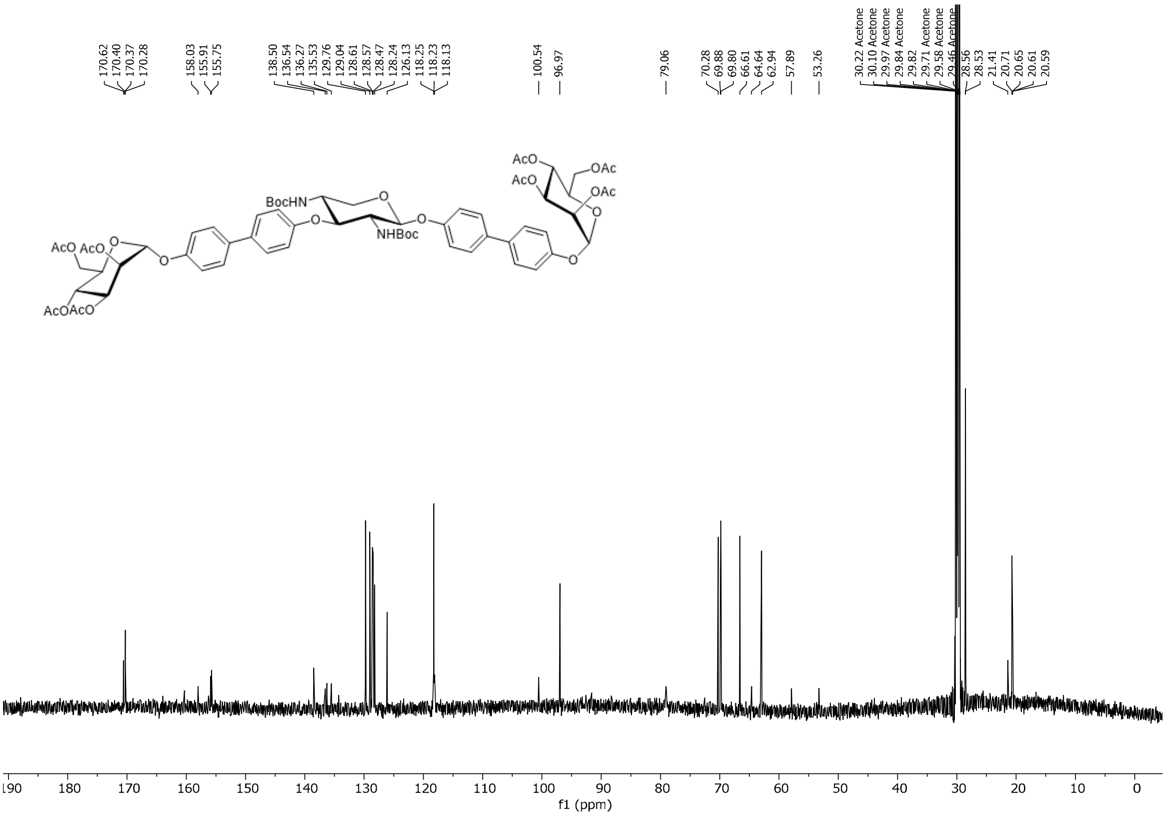

Figure S4. ^{13}C NMR spectrum of **3** (125 MHz, acetone-d$_6$, 298 K).

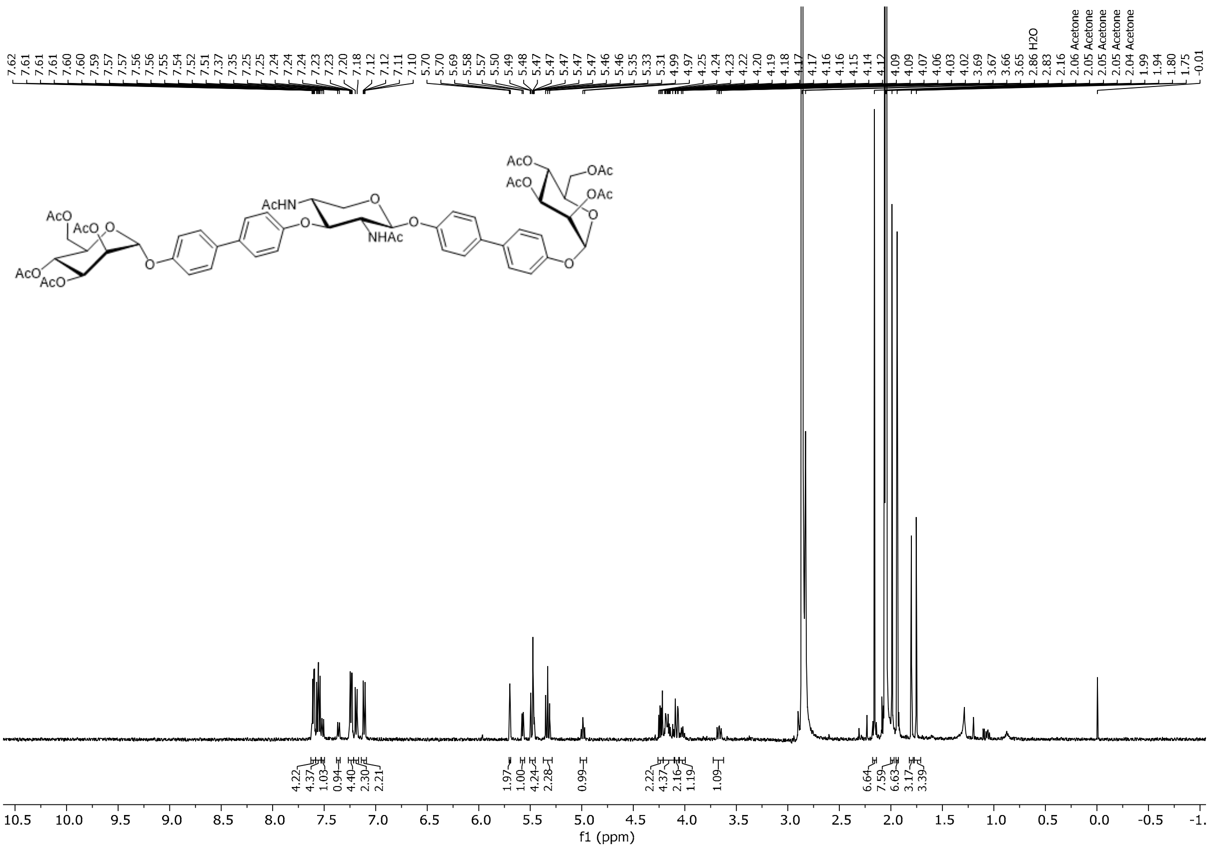

Figure S5. ^{1}H NMR spectrum of **4** (500 MHz, acetone-d_6, 298 K).

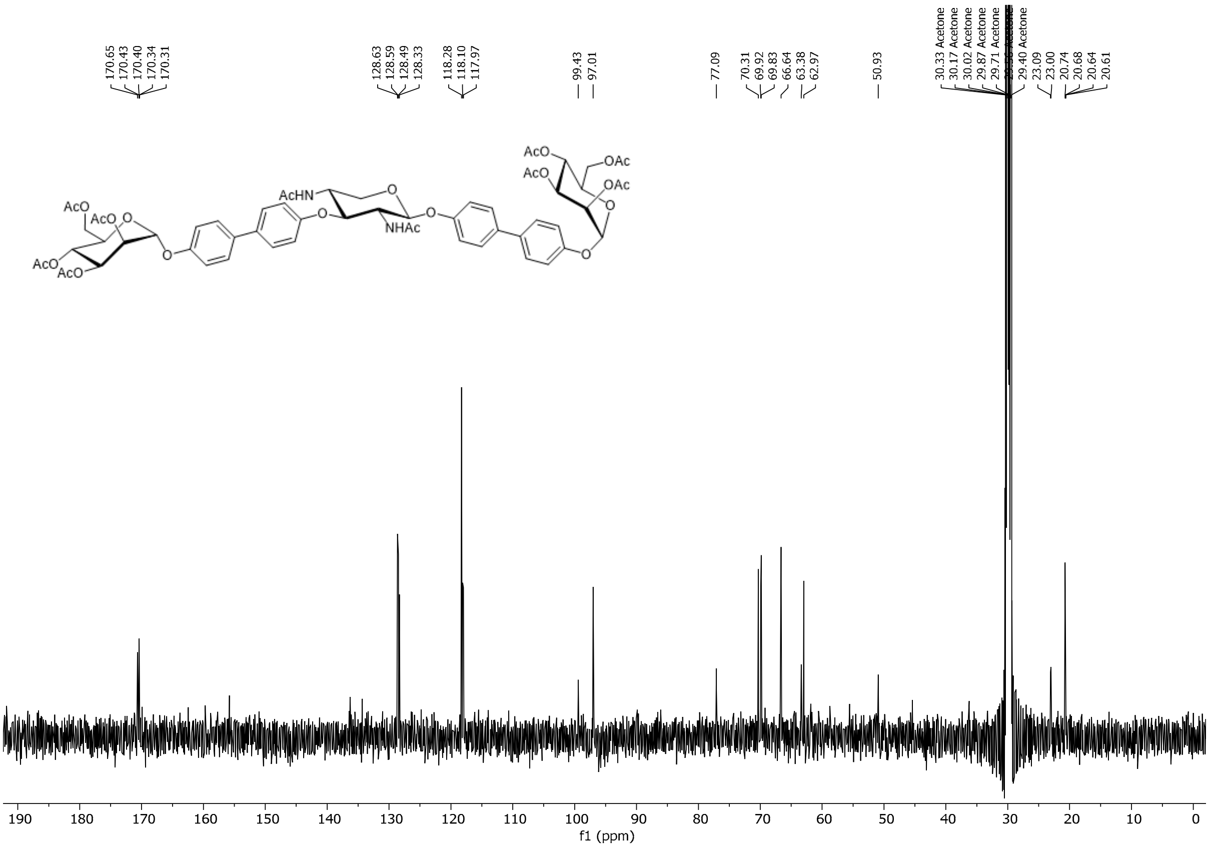

Figure S6. ^{13}C NMR spectrum of **4** (125 MHz, acetone-d_6, 298 K).

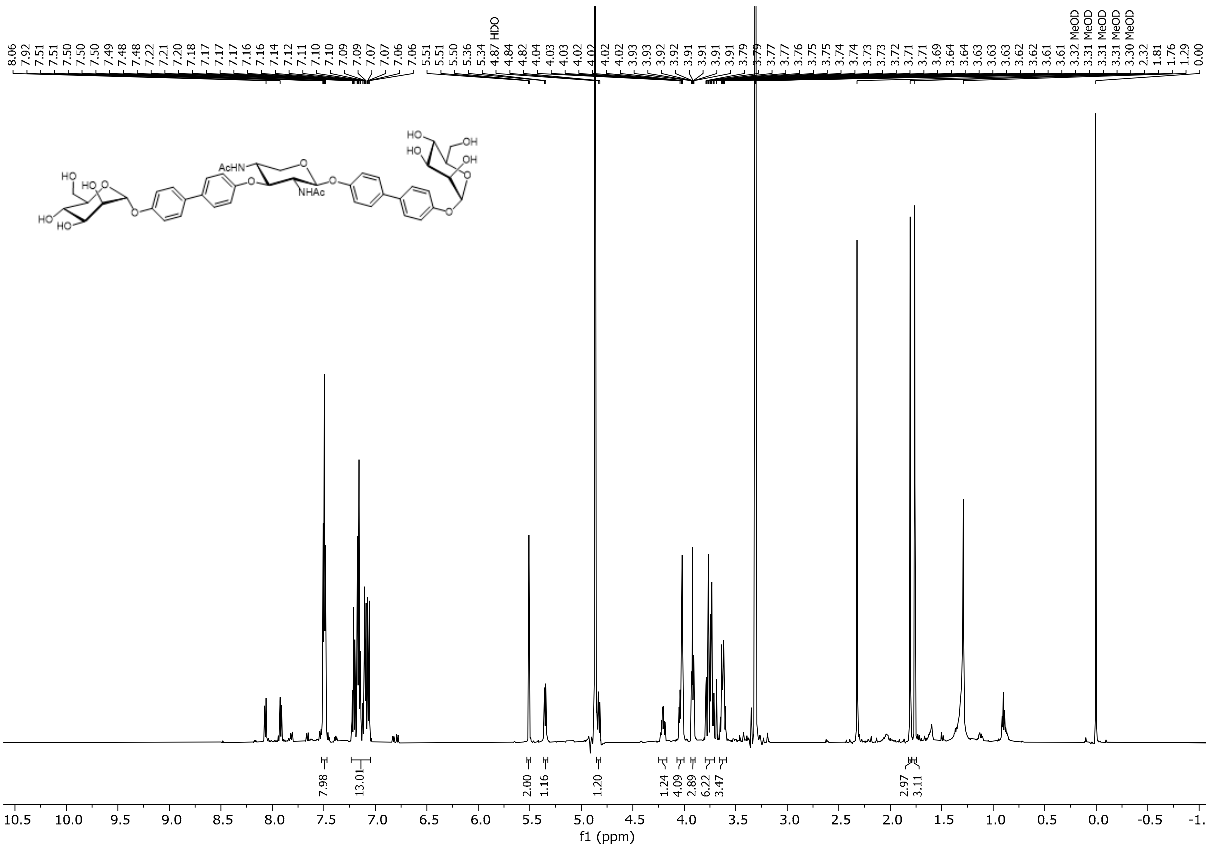

Figure S7. ^{1}H NMR spectrum of **5** (600 MHz, MeOD, 298 K).

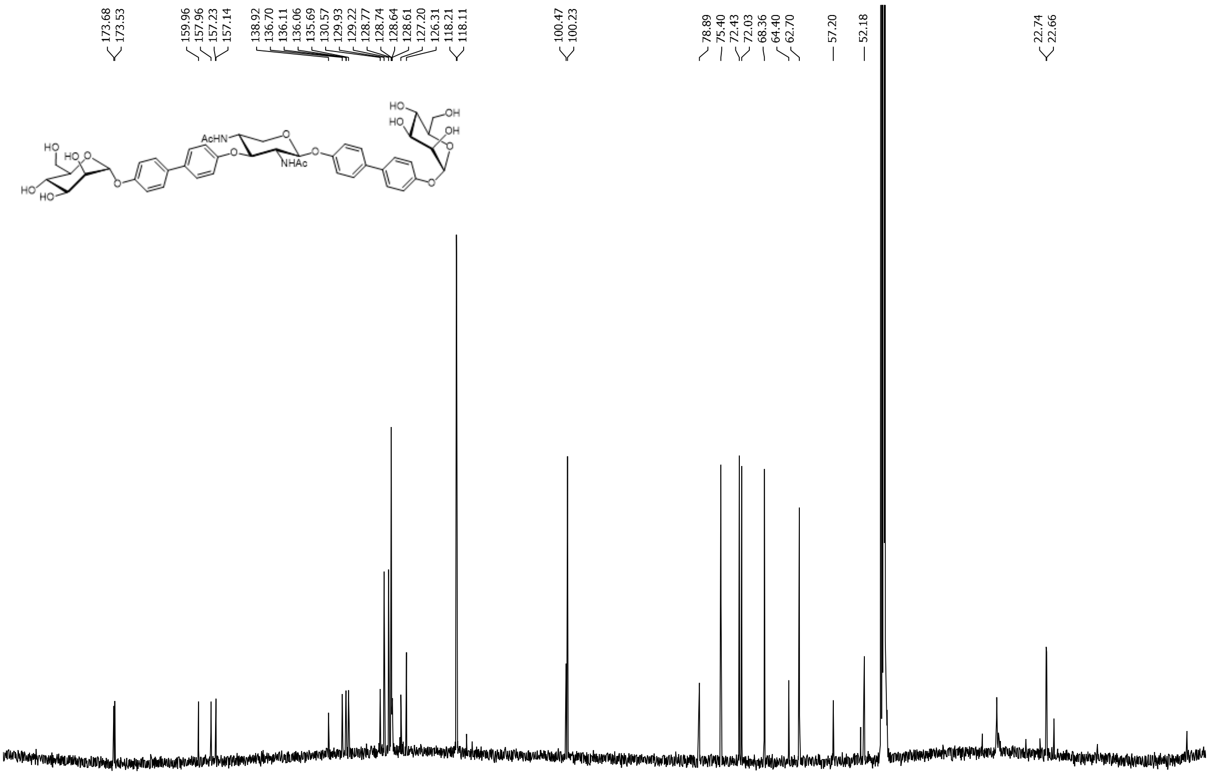

Figure S8. ^{13}C NMR spectrum of **5** (125 MHz, MeOD, 298 K).

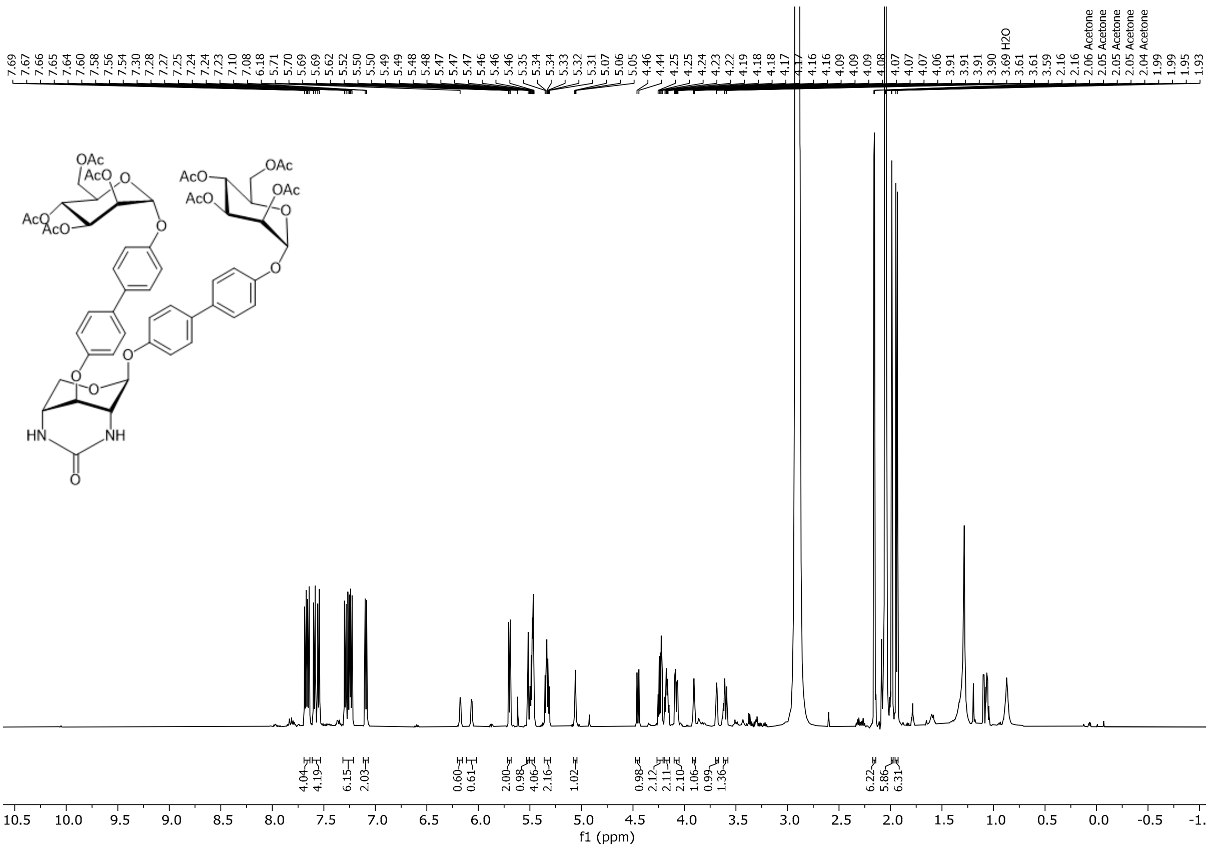

Figure S9. ^{1}H NMR spectrum of **6** (500 MHz, aceton-d_6, 298 K).

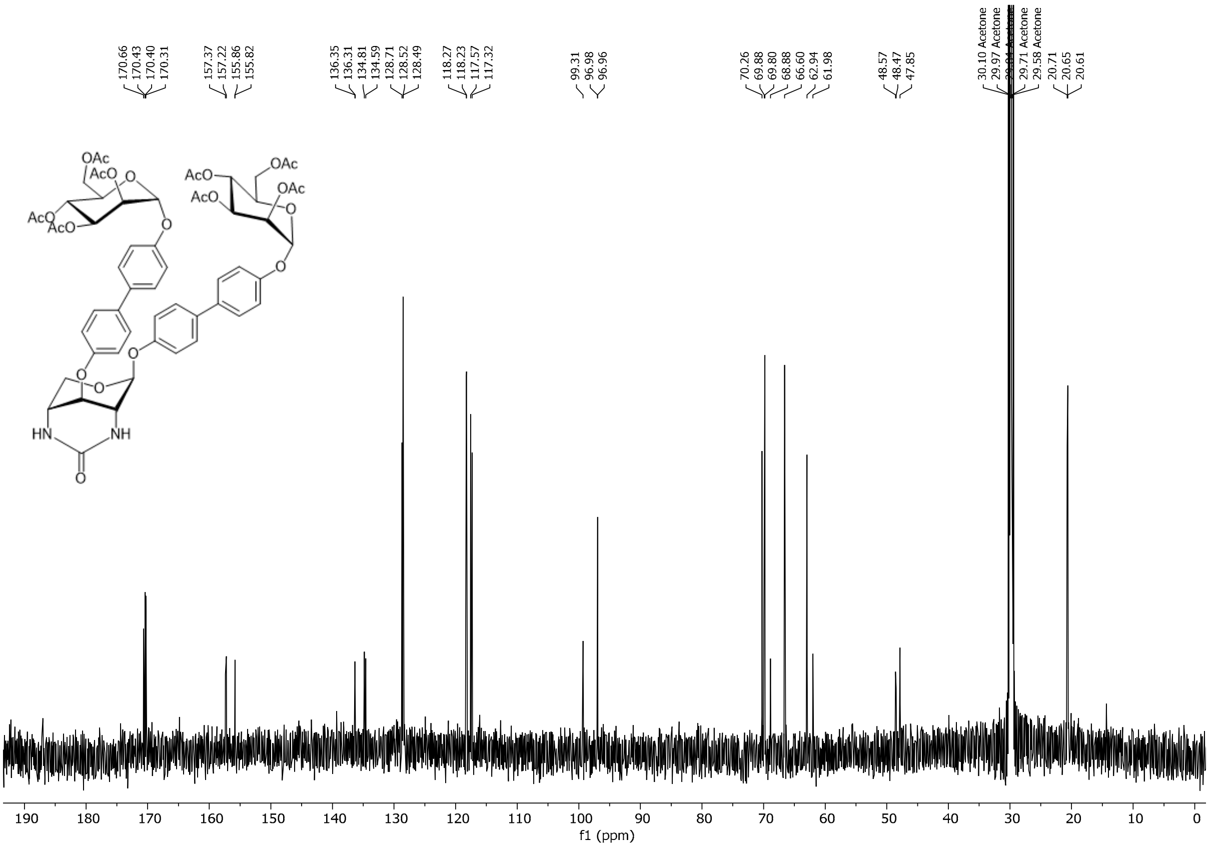

Figure S10. ^{13}C NMR spectrum of **6** (125 MHz, aceton-d6, 298 K).

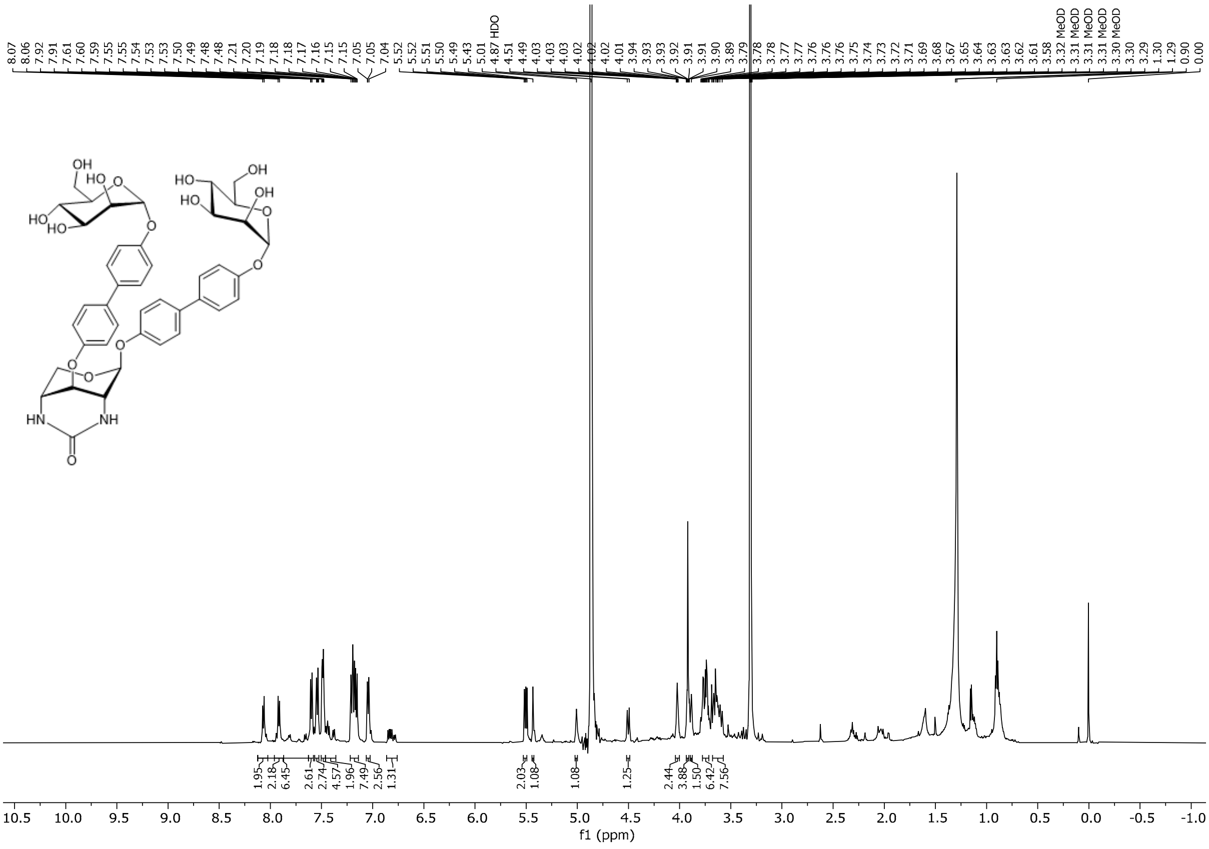

Figure S11. ^{1}H NMR spectrum of **7** (600 MHz, MeOD, 298 K).

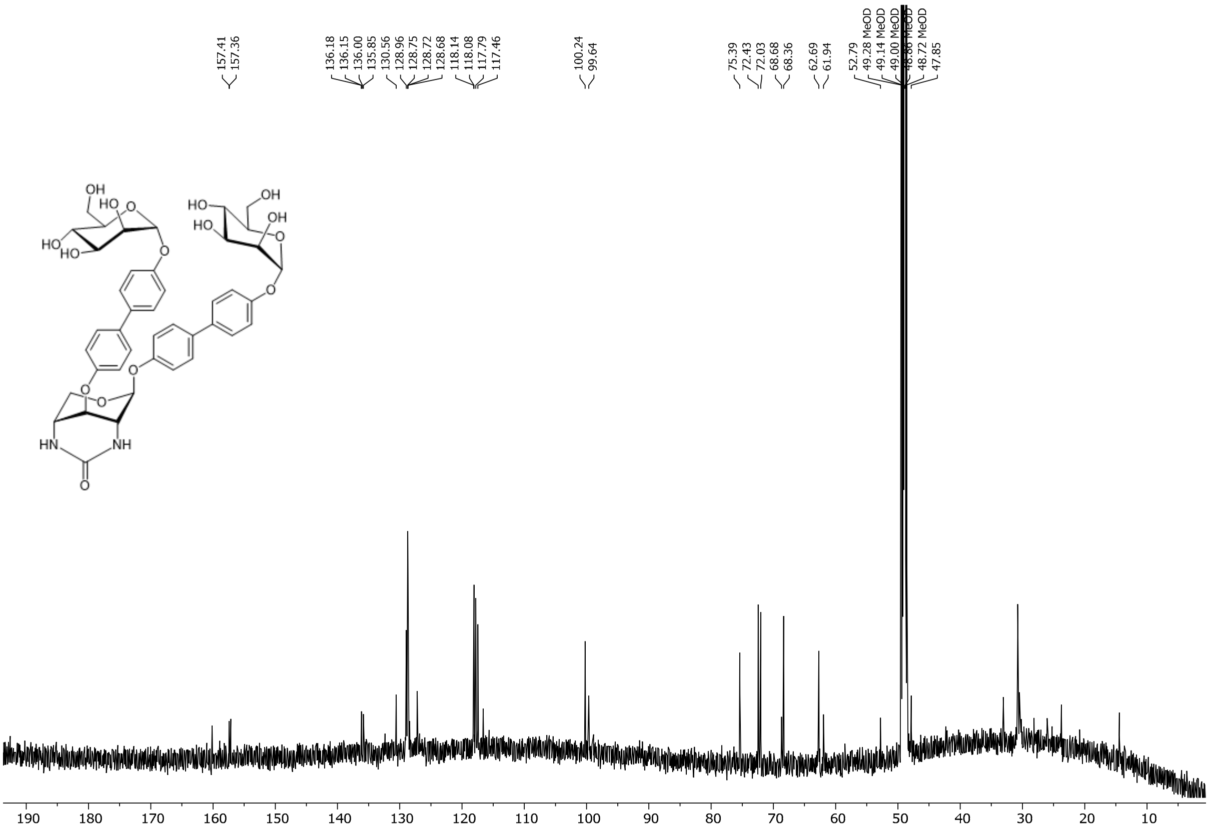

Figure S12. ^{13}C NMR spectrum of **7** (125 MHz, MeOD, 298 K).

References

1. Reisner, A.; Haagensen, J.A.J.; Schembri, M.A.; Zechner, E.L.; Molin, S. Development and Maturation of Escherichia Coli K-12 Biofilms. *Mol. Microbiol.* **2003**, *48*, 933–946, doi:10.1046/j.1365-2958.2003.03490.x.
2. Hartmann, M.; K. Horst, A.; Klemm, P.; K. Lindhorst, T. A Kit for the Investigation of Live Escherichia Coli Cell Adhesion to Glycosylated Surfaces. *Chem. Commun.* **2010**, *46*, 330–332, doi:10.1039/B922525K.
3. Schrödinger Release 2021-1: Maestro, Schrödinger, LLC, New York, NY, 2021.
4. Schrödinger Release 2024-2: Maestro, Schrödinger, LLC, New York, NY, 2024.
5. Schrödinger Release 2021-1: Desmond Molecular Dynamics System, D. E. Shaw Research, New York, NY, 2021. Maestro-Desmond Interoperability Tools, Schrödinger, New York, NY, 2021.
6. Mark, P.; Nilsson, L. Structure and Dynamics of the TIP3P, SPC, and SPC/E Water Models at 298 K. *J. Phys. Chem. A* **2001**, *105*, 9954–9960, doi:10.1021/jp003020w.
7. Lu, C.; Wu, C.; Ghoreishi, D.; Chen, W.; Wang, L.; Damm, W.; Ross, G.A.; Dahlgren, M.K.; Russell, E.; Von Bargen, C.D.; et al. OPLS4: Improving Force Field Accuracy on Challenging Regimes of Chemical Space. *J. Chem. Theory Comput.* **2021**, *17*, 4291–4300, doi:10.1021/acs.jctc.1c00302.
8. Schönemann, W.; Cramer, J.; Mühlethaler, T.; Fiege, B.; Silbermann, M.; Rabbani, S.; Dätwyler, P.; Zihlmann, P.; Jakob, R.P.; Sager, C.P.; et al. Improvement of Aglycone π-Stacking Yields Nanomolar to Sub-Nanomolar FimH Antagonists. *ChemMedChem* **2019**, *14*, 749–757, doi:10.1002/cmdc.201900051.
9. Schrödinger Release 2024-2: Glide, Schrödinger, LLC, New York, NY, 2024.
10. Schrödinger Release 2024-2: Prime, Schrödinger, LLC, New York, NY, 2024.
